# SUCNR1 regulates insulin secretion and glucose elevates the succinate response in people with prediabetes

**DOI:** 10.1172/JCI173214

**Published:** 2024-05-07

**Authors:** Joan Sabadell-Basallote, Brenno Astiarraga, Carlos Castaño, Miriam Ejarque, Maria Repollés-de-Dalmau, Ivan Quesada, Jordi Blanco, Catalina Núñez-Roa, M-Mar Rodríguez-Peña, Laia Martínez, Dario F. De Jesus, Laura Marroquí, Ramon Bosch, Eduard Montanya, Francesc X. Sureda, Andrea Tura, Andrea Mari, Rohit N. Kulkarni, Joan Vendrell, Sonia Fernández-Veledo

**Affiliations:** 1 Unitat de Recerca, Hospital Universitari Joan XXIII, Institut d’Investigació Sanitària Pere Virgili, Tarragona, Spain.; 2 CIBER de Diabetes y Enfermedades Metabólicas Asociadas (CIBERDEM), Instituto de Salud Carlos III, Madrid, Spain.; 3 Universitat Rovira i Virgili, Tarragona, Spain.; 4 Islet Cell and Regenerative Biology, Joslin Diabetes Center, Department of Medicine, Beth Israel Deaconess Medical Center, Harvard Stem Cell Institute, Harvard Medical School, Boston, Massachusetts, USA.; 5 Instituto de Investigación, Desarrollo e Innovación en Biotecnología Sanitaria de Elche (IDiBE), Universidad Miguel Hernández de Elche, Alicante, Spain.; 6 Histological, Cytological and Digitization Studies Platform, Pathology Department, Hospital Verge de la Cinta, Tortosa, Spain.; 7 Hospital Universitari de Bellvitge, Bellvitge Biomedical Research Institute (IDIBELL), and Universitat de Barcelona, Barcelona, Spain.; 8 Institute of Neuroscience, National Research Council, Padua, Italy.

**Keywords:** Endocrinology, Metabolism, Beta cells, G protein&ndash;coupled receptors, Insulin

## Abstract

Pancreatic β cell dysfunction is a key feature of type 2 diabetes, and novel regulators of insulin secretion are desirable. Here, we report that succinate receptor 1 (SUCNR1) is expressed in β cells and is upregulated in hyperglycemic states in mice and humans. We found that succinate acted as a hormone-like metabolite and stimulated insulin secretion via a SUCNR1-Gq-PKC–dependent mechanism in human β cells. Mice with β cell–specific *Sucnr1* deficiency exhibited impaired glucose tolerance and insulin secretion on a high-fat diet, indicating that SUCNR1 is essential for preserving insulin secretion in diet-induced insulin resistance. Patients with impaired glucose tolerance showed an enhanced nutrition-related succinate response, which correlates with the potentiation of insulin secretion during intravenous glucose administration. These data demonstrate that the succinate/SUCNR1 axis is activated by high glucose and identify a GPCR-mediated amplifying pathway for insulin secretion relevant to the hyperinsulinemia of prediabetic states.

## Abstract 2

SUCNR1, expressed in β cells and upregulated in hyperglycemia, is essential for maintaining insulin secretion in diet-induced insulin resistance and prediabetic states.

## Graphical abstract

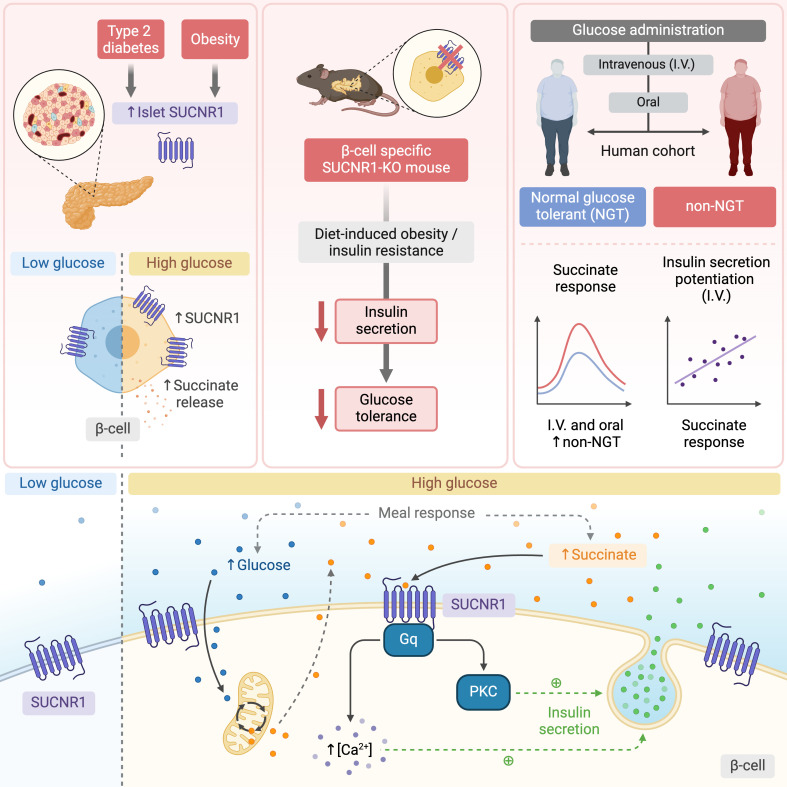

## Introduction

Insulin secretion by pancreatic β cells is crucial for blood glucose homeostasis and is tightly controlled by a complex network of hormones, nutrients, and neurotransmitters. Impaired β cell function worsened by the compensatory hyperinsulinemia associated with insulin-resistant states is a key contributing factor of type 2 diabetes (T2D), a major chronic health concern primarily linked to obesity (1–4). Two different pathways are known to interact in β cells to ensure insulin secretion in response to glucose stimulation. The well-known “triggering” pathway involves the mitochondrial metabolism of glucose, which induces the closure of ATP-sensitive potassium channels and the activation of voltage-gated Ca^2+^ channels, ultimately triggering the exocytosis of insulin granules (5). However, a second or “metabolic amplifying” pathway is necessary for proper insulin secretion response, involving the external replenishment of the Krebs, or tricarboxylic acid (TCA), cycle via anaplerosis (6), and is distinct to the neurohormonal amplification pathways (e.g., incretin system) that are related to the activation of G protein–coupled receptors (GPCRs) (7). In addition to this dual and hierarchical control of insulin secretion, emerging evidence points to important roles for anaplerotic TCA cycle substrates that act as second messengers in the cytosol to drive insulin granule exocytosis (8). Within this framework, energy metabolites are increasingly recognized as signaling molecules (9).

In recent years, the TCA cycle substrate succinate has become the poster child for the concept that metabolites can have functions beyond energy metabolism. Cytosolic succinate accumulation occurs largely in response to aerobic glycolysis, breakdown of the TCA cycle, and anaplerosis (10). Succinate can also be released into the extracellular space by specific plasma membrane transporters (11, 12), where it behaves akin to classical hormones and cytokines through engagement with succinate receptor 1 (SUCNR1) (10). Additionally, it has long been known that cell-permeable succinate esters are approximately one-third as potent as glucose at stimulating insulin release, and it is hypothesized that the insulinotropic properties of succinate are based on its capacity to increase the production of mevalonate and NADPH (13–16). Succinate might also act as a stimulus-coupling secondary signal for proinsulin biosynthesis independently of insulin secretion (17, 18). Importantly, succinate has been described as the TCA cycle metabolite more closely associated with second-phase insulin secretion (19). Nevertheless, the extramitochondrial and extracytosolic roles of succinate in insulin secretion are unexplored.

SUCNR1 expression has been observed in the pancreas (20, 21) and in human islets (22), indicating a potential instructive role for the succinate/SUCNR1 axis in islet physiology. Elevated circulating levels of succinate and SUCNR1 activation have been traditionally ascribed to hypoxia, tissue damage, and inflammatory processes (23). Correspondingly, SUCNR1 signaling has been primarily linked to pathophysiology, including metabolic disorders such as obesity, diabetes, and non-alcoholic fatty liver disease (24–31). Nonetheless, previous research has also described a transient rise in extracellular succinate during physiological processes, including exercise (12, 32), with which it is involved in muscle innervation and matrix remodeling via SUCNR1 (12). Also, we recently demonstrated that dynamic succinate regulation occurs during fasted-to-fed transition, which is potentiated when nutrients are sensed by the intestine but is disturbed in unhealthy metabolic states (33). Therefore, the initial view of SUCNR1 as an exclusive sensor for local damage is being reshaped in the light of recent observations of succinate as a systemic metabolic signal.

SUCNR1 can signal through cAMP- or Ca^2+^-dependent pathways (34–36), which are well-known regulators of insulin secretion in β cells (37). Notably, global-*Sucnr1*-knockout mice fed a high-fat diet (HFD) show defective insulin secretion and glucose intolerance, without significant changes in β cell mass (25), raising suspicion of its role as a regulator of β cell function. Here, we adopted a comprehensive and integrative translational approach to investigate the impact of the succinate/SUCNR1 axis on the β cell function. Our investigation using a unique pancreatic β cell–specific knockout mouse model and in vitro analyses demonstrates that the succinate/SUCNR1 axis serves a glucose-dependent amplifying transduction pathway enhancing insulin release. Furthermore, we find that succinate functions as an incretin-like metabolite, which significantly potentiates insulin secretion in hyperglycemia in humans.

## Results

### Pancreatic β cells express SUCNR1.

**Figure 1:**
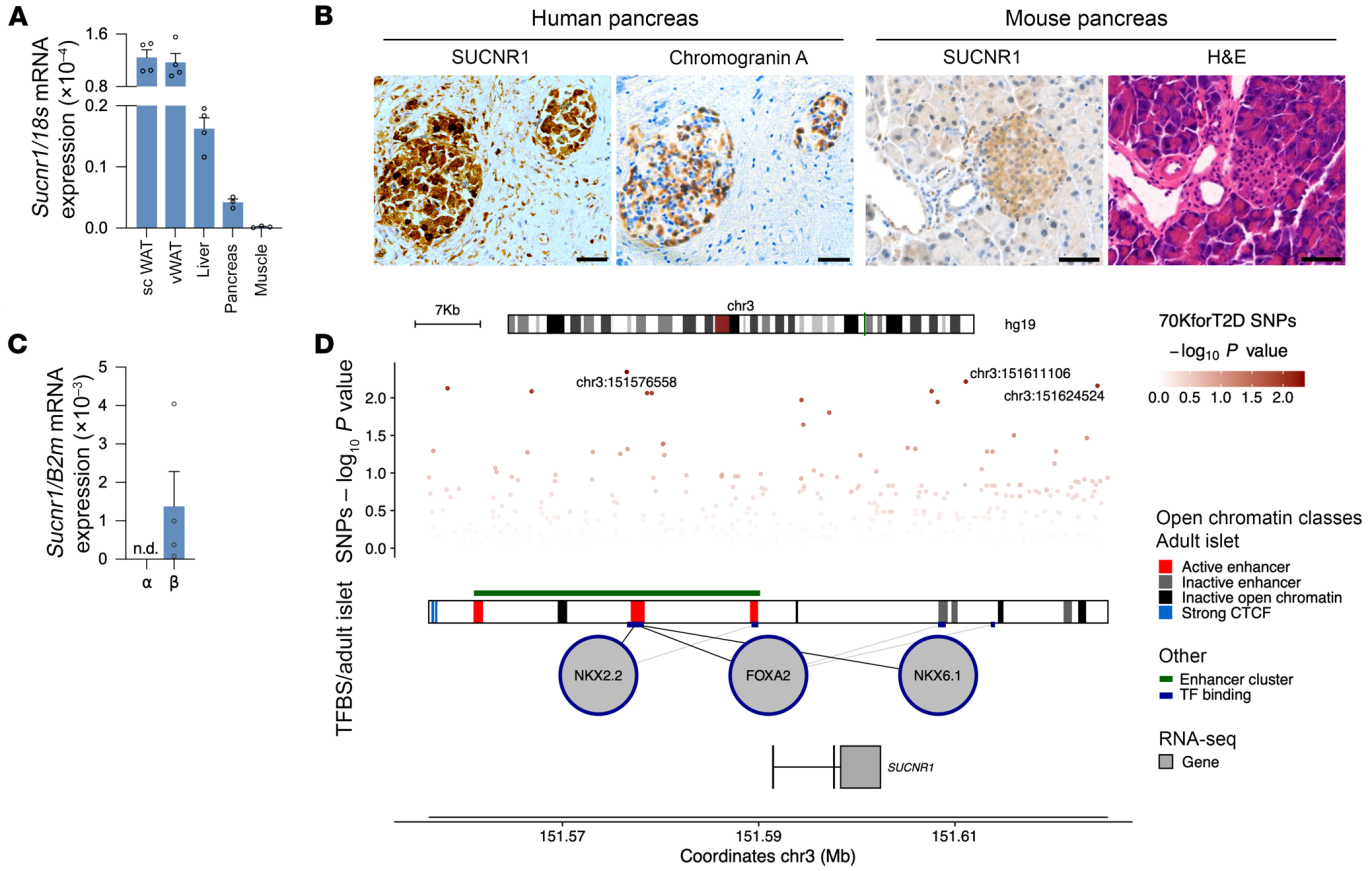
SUCNR1 is expressed in islets and β cells. (**A**) *Sucnr1* mRNA levels analyzed in subcutaneous white adipose tissue (scWAT), visceral WAT tissue (vWAT), and liver, pancreas, and muscle tissue from male mice by quantitative PCR (*n* = 3–4). (**B**) Immunohistochemical (IHC) staining of SUCNR1 in male human and male mouse pancreas sections, and chromogranin A IHC staining or H&E staining. Scale bars: 50 μm. (**C**) Analysis of *Sucnr1* mRNA expression in α and β cells isolated by FACS from male rat islets (*n* = 4). (**D**) In silico study of *SUCNR1* gene expression regulation by genomic sequences and specific human adult islet transcriptional factors, and single-nucleotide polymorphisms (SNPs) associated with T2D localized within or surrounding the *SUCNR1* locus. TFBS, transcription factor binding site. Data are presented as mean ± SEM.

We first analyzed *Sucnr1* mRNA levels in metabolic tissues from mice (Figure 1A), confirming *Sucnr1* expression in the total pancreas, albeit at low levels compared with white adipose tissue and liver, in agreement with previous reports (20, 21, 38). Immunohistochemical staining of pancreas sections revealed a higher SUCNR1 protein abundance in islets than in exocrine tissue in both human and mouse samples (Figure 1B). To gain insight into the cell type–specific expression of SUCNR1 in pancreatic islets, we examined its mRNA levels in the 2 major cell populations: α and β cells (39). Results showed that *Sucnr1* was specifically expressed in β cells from rat islets (Figure 1C). Gene expression regulation differs across tissues, and particular factors may influence *SUCNR1* expression in human islets. In silico analysis of the human *SUCNR1* gene using the Islet Regulome Browser (40) identified NKX2.2, FOXA2, and NKX6.1 as putative regulators of *SUCNR1* expression, which are key transcription factors for β cell identity and function (41–43). Bioinformatics analysis also revealed *SUCNR1* expression in a published RNA-Seq library from human islets (44). Finally, the *SUCNR1* locus and its surrounding regions contained several single-nucleotide polymorphisms identified in patients with T2D in a genome-wide association study data set (45) (Figure 1D).

### SUCNR1 expression in islets is regulated in human obesity and T2D.

**Figure 2:**
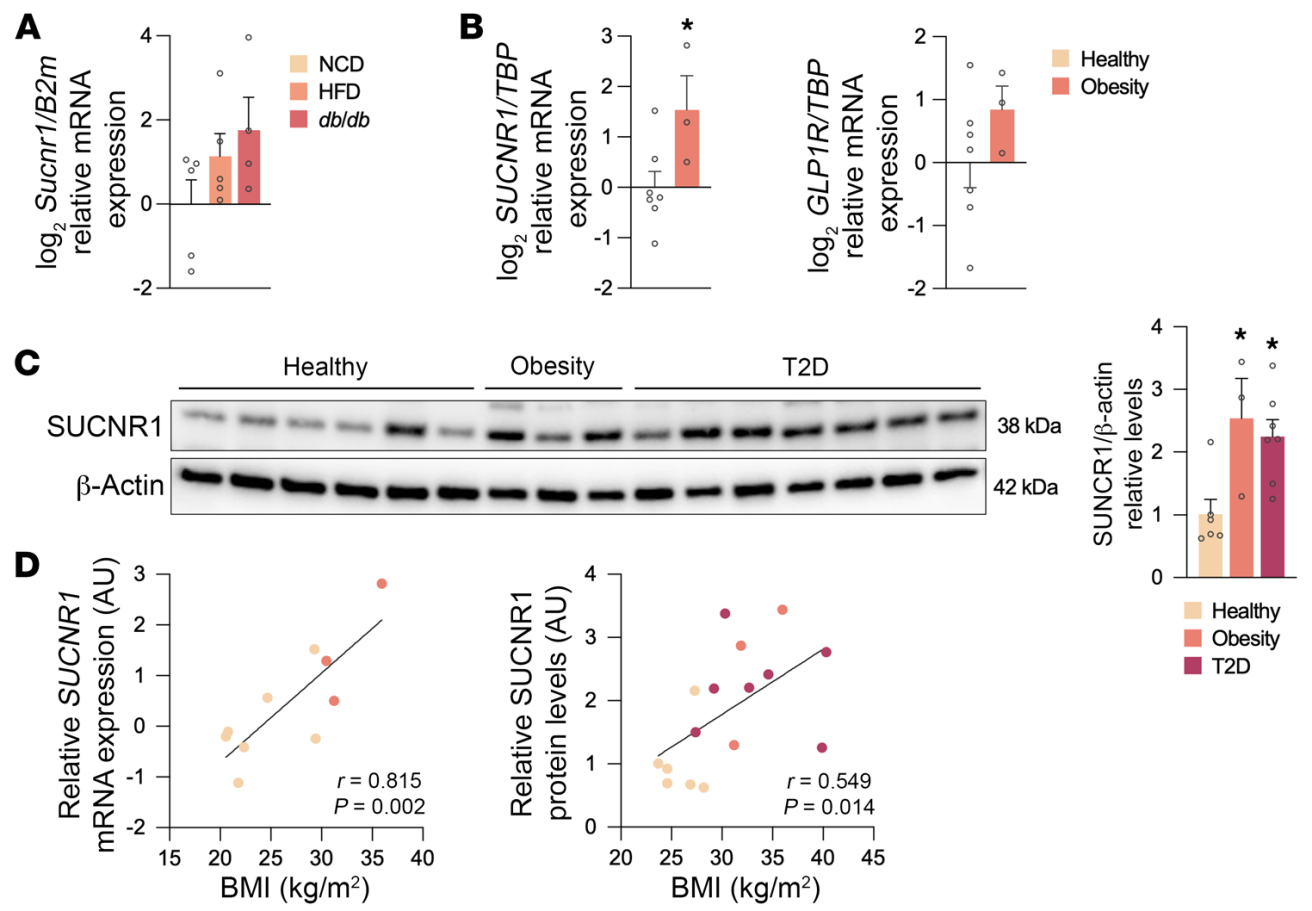
SUCNR1 levels in islets are dysregulated in obesity and T2D. (**A**) *Sucnr1* mRNA expression in the entire pancreas of wild-type mice fed normal chow diet (NCD) and high-fat diet (HFD) and *db/db* mice on NCD (*n* = 4–5). (**B**) *SUCNR1* and *GLP1R* mRNA expression in islets from healthy donors (*n* = 7) and donors with obesity (*n* = 3). (**C**) SUCNR1 protein levels in human islet lysates from healthy donors (*n* = 6) and donors with obesity (*n* = 3) and T2D (*n* = 7). (**D**) Linear correlations between the BMI of donors and SUCNR1 mRNA (*n* = 10) and protein (*n* = 16) expression. Data are presented as mean ± SEM. *P < 0.05 vs. control (Student’s *t* test in **A** and **B**, Kruskal-Wallis test with Dunn’s test for multiple comparisons in **C**, or Pearson’s correlation coefficient in **D**).

To evaluate whether *Sucnr1* is regulated in the pancreas in different metabolic states, we first assessed its expression in total pancreas tissue from rodent models of obesity and T2D. We observed a trend for elevated expression in diet-induced obese (DIO) mice and a further trend increase in expression in diabetic (*db/db*) mice (Figure 2A). Comparable but significant results were obtained in human pancreatic islets from patients with obesity/T2D in 2 independent cohorts, without significant changes in the levels of *GLP1R* (Figure 2B; donor information in Supplemental Table 1; supplemental material available online with this article; https://doi.org/10.1172/JCI173214DS1). These results were confirmed at the protein level in human islet lysates (Figure 2C; donor information in Supplemental Table 2). In addition, we found a positive correlation between *SUCNR1* mRNA/protein levels in islets and the body mass index (BMI) of the human donors (Figure 2D). Overall, these data indicate that islet SUCNR1 is associated with changes in metabolic status, implying a potential role of this receptor in the pathophysiology of metabolic disorders and T2D.

### β Cells sense extracellular succinate and release insulin in a SUCNR1-Gq-PKC–dependent mechanism.

**Figure 3:**
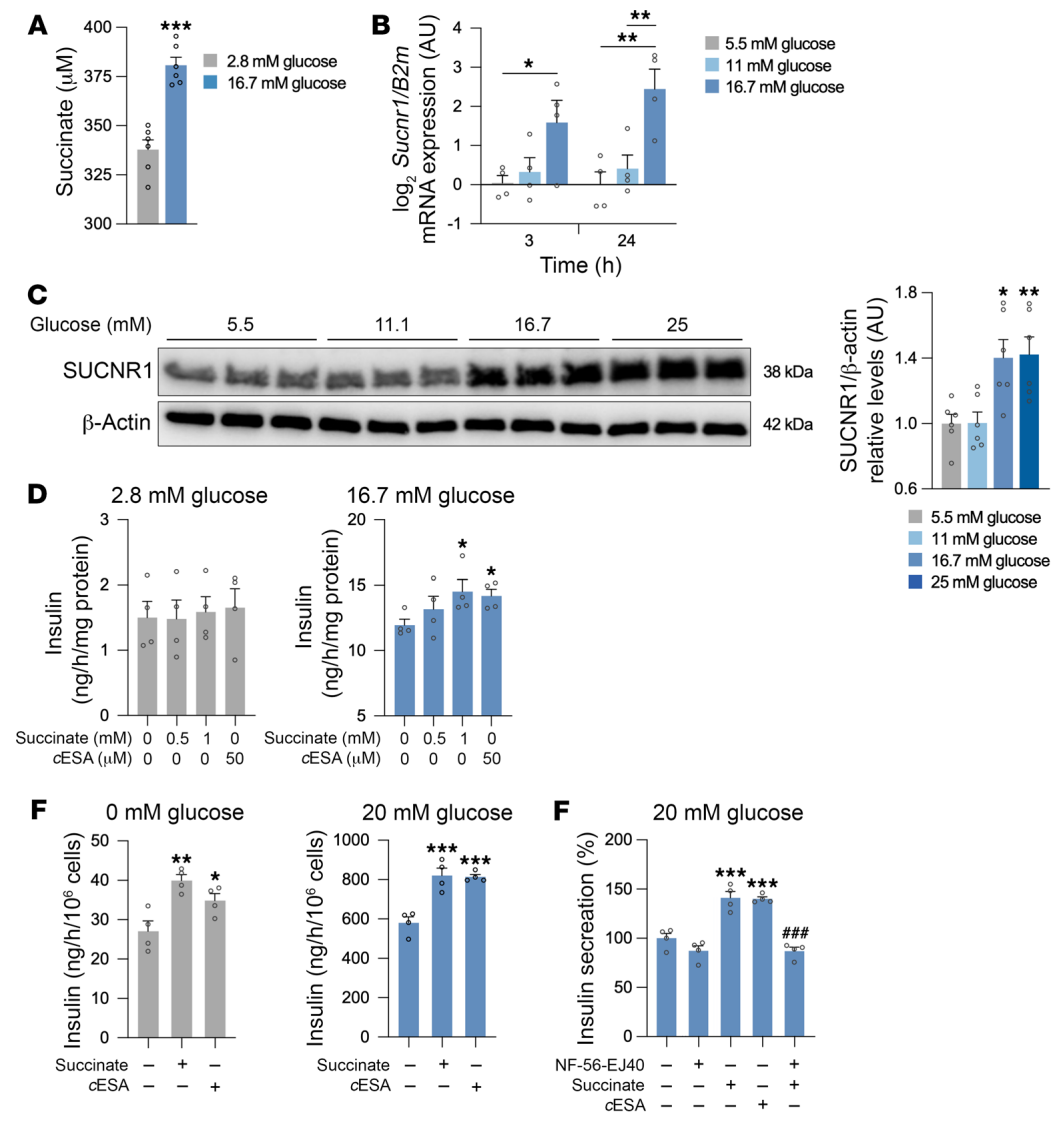
The succinate/SUCNR1 axis enhances glucose-stimulated insulin secretion in β cells. (**A**) Succinate quantification in the conditioned medium (CM) of MIN6 cells cultured in low- or high-glucose conditions (*n* = 6). (**B**) *Sucnr1* mRNA expression in MIN6 cells stimulated with different concentrations of glucose for 3 or 24 hours (*n* = 4). (**C**) SUCNR1 protein levels in EndoC-βH1 cells stimulated with different concentrations of glucose for 24 hours (*n* = 6). (**D**) Insulin quantification in the CM of MIN6 cells stimulated with succinate or *cis*-epoxysuccinic acid (*c*ESA) at 2.8 mM or 16.7 mM glucose (*n* = 4). (**E**) Insulin secretion in glucose-stimulated insulin secretion assays in EndoC-βH5 cells stimulated with 500 μM succinate or 50 μM *c*ESA at 0 mM or 20 mM glucose, determined in the CM by ELISA (*n* = 4). (**F**) Insulin secretion in EndoC-βH5 cells incubated with a human-specific SUCNR1 antagonist (1 μM NF-56-EJ40) and 500 μM succinate or 50 μM *c*ESA (*n* = 4). Data are presented as mean ± SEM. *P < 0.05, **P < 0.01, ***P < 0.001 vs. basal conditions; ^###^*P* < 0.001 vs. succinate (Student’s *t* test in **A**, ANOVA with Dunnett’s test for multiple comparisons in **B**–**E**, or ANOVA with Tukey’s test for multiple comparisons in **F**).

We next explored a potential link between hyperglycemia and the succinate/SUCNR1 axis in β cells. Glucose catabolism increases the intracellular levels of succinate in β cells through pyruvate oxidation via the TCA cycle (46). We observed that high-glucose exposure of the mouse-derived β cell line MIN6 stimulated succinate release to the extracellular medium (Figure 3A). As pancreatic β cells can regulate their behavior in response to glucose, we analyzed *Sucnr1* expression in MIN6 cells cultured at different glucose concentrations, and found that glucose increased *Sucnr1* expression in a dose-dependent manner (Figure 3B). This result was confirmed at the protein level in the human β cell line EndoC-βH1 cultured at increasing glucose concentrations (Figure 3C).

Our findings that β cells might increase their sensitivity to extracellular succinate by increasing SUCNR1 expression in response to glucose in a dose-dependent manner prompted us to investigate whether SUCNR1 activation impacts insulin secretion. We observed that extracellular succinate significantly potentiated (20% increase) glucose-stimulated insulin secretion (GSIS) in MIN6 cells (Figure 3D). Even though succinate in culture medium takes an anionic form, and as such is widely considered as non-cell-permeable (16), we also used the synthetic SUCNR1 agonist *cis*-epoxysuccinic acid (*c*ESA), which has a lower EC_50_ compared with succinate (47), to exclude any intracellular effects of succinate induced by its uptake into cells via dicarboxylic acid transporters (48, 49). Results showed that *c*ESA similarly augmented insulin secretion under high glucose (Figure 3D).

The effect of succinate as an extracellular insulin secretagogue was confirmed in EndoC-βH5 cells, which, compared with other commonly used human β cell lines, do not present limitations regarding the expression of some GPCRs (i.e., GLP-1R) (50, 51). EndoC-βH5 cells showed an increase in insulin secretion when SUCNR1 was activated by extracellular succinate or *c*ESA under conditions of no glucose (1.5-fold or 1.3-fold increase, respectively) and high-glucose conditions (1.4-fold increase for both) (Figure 3E). To further demonstrate that the effect of succinate on insulin release is dependent on its extracellularly driven signaling properties, we cotreated cells with the human-specific SUCNR1 antagonist NF-56-EJ40 (52) during the GSIS assay. Acute receptor antagonism completely blocked succinate-induced insulin secretion in the presence of high glucose (Figure 3F), confirming that the insulinotropic effect of extracellular succinate is dependent on SUCNR1.

**Figure 4:**
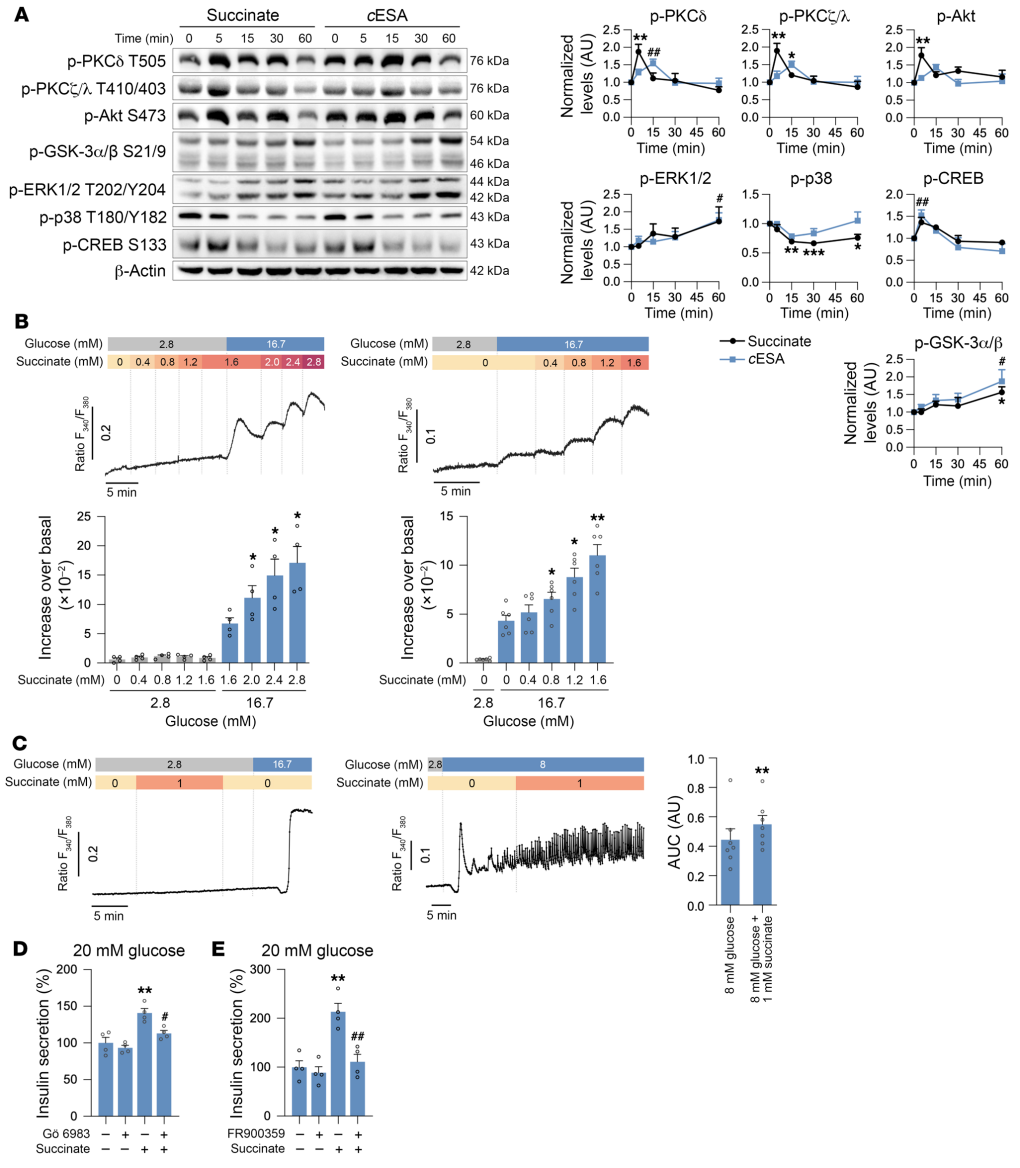
SUCNR1 activation in β cells induces proximal signaling and Ca^2+^ mobilization, and is dependent on Gq and PKC pathways in β cells. (**A**) Western blot analysis of several phosphoproteins in MIN6 cells stimulated with 500 μM succinate or 50 μM *c*ESA at different time points (*n* = 4–5). (**B**) Ca^2+^ mobilization in MIN6 cells stimulated with glucose and succinate assessed by Fura-2 AM fluorometric ratio. Left: Representative trace and quantification of the response to sequential increases in succinate concentration before and after high glucose exposure (*n* = 4). Right: Representative trace and quantification of the response to succinate concentrations elevated similarly after high glucose exposure (*n* = 6). (**C**) Intracellular Ca^2+^ mobilization in perifused islets from wild-type C57BL/6 male mice assessed by Fura-2 AM fluorometric ratio. Left: Representative trace of 1 mM succinate’s effect on intracellular Ca^2+^ mobilization at a basal glucose concentration (2.8 mM), followed by exposure to 16.7 mM glucose (*n* = 7 islets from 3 mice). Right: Representative trace and quantification of 1 mM succinate’s effect on intracellular Ca^2+^ mobilization at 8 mM glucose (*n* = 7 islets from 2 mice). (**D**) Insulin secretion in EndoC-βH5 cells incubated with 500 μM succinate and 1 μM of PKC inhibitor Gö 6983 (*n* = 4). (**E**) Insulin secretion in EndoC-βH5 cells incubated with 500 μM succinate and 1 μM of Gq inhibitor FR900359 (*n* = 4). Data are presented as mean ± SEM. *P < 0.05, **P < 0.01, ***P < 0.001, succinate vs. basal condition; ^#^*P* < 0.05, ^##^*P* < 0.01, *c*ESA vs. basal condition or an inhibitor vs. succinate (ANOVA with Dunnett’s test for multiple comparisons in **A** and **B**, paired Student’s *t* test in **C**, or ANOVA with Tukey’s test for multiple comparisons in **D** and **E**).

We next investigated the underlying mechanism of insulin release by SUCNR1 in β cells. While SUCNR1 transduction pathways remain largely elusive (53), it is increasingly recognized that they are cell and context dependent. We thus challenged MIN6 cells with succinate or *c*ESA and analyzed several canonical proteins that might integrate GPCR signal transduction. Results of Western blotting revealed a succinate-dependent increase in the phosphorylated forms of AKT and the novel and atypical PKCs, PKCδ and PKCζ, respectively, which was accompanied by the differential phosphorylation of ERK1/2 and p38 MAPK (Figure 4A). Concomitant with this activation, there was an increase in phosphorylated CREB and phosphorylated (inactive) GSK-3α/β. As β cells are sensitive to changes in [Ca^2+^], we also assessed Ca^2+^ mobilization in MIN6 cells upon SUCNR1 activation using a fluorometric assay to measure real-time changes in intracellular Ca^2+^ ([Ca^2+^]_i_). We first challenged low-glucose-cultured MIN6 cells with additive doses of succinate, observing no changes in [Ca^2+^]_i_. However, when glucose in the culture medium was increased to 16.7 mM, [Ca^2+^]_i_ was progressively mobilized with increasing additions of succinate (ANOVA after glucose addition *P* = 0.0126). As cells were exposed to increasing succinate levels before high-glucose stimulation, we studied the succinate effect on Ca^2+^ mobilization immediately after glucose addition. Notably, we also observed a significant glucose-dependent succinate dose-response effect on [Ca^2+^]_i_ in MIN6 cells at lower extracellular succinate doses, which reached significance at 800 μM succinate (ANOVA after glucose addition *P* = 0.0013) (Figure 4B). Expanding on our analysis of MIN6 cells, we examined the influence of succinate on perifused mouse pancreatic islets. Similarly, succinate exhibited no noticeable impact on [Ca^2+^]_i_ when islets were exposed to non-stimulatory glucose concentrations, such as 2.8 mM. Since glucose elicits submaximal to maximal Ca^2+^ responses at 16.7 mM in mouse islets (54), which could complicate further assessments of Ca^2+^ effects, we addressed the impact of succinate on glucose-induced Ca^2+^ signals at 8 mM. As shown in Figure 4C, 8 mM glucose elicited the characteristic initial Ca^2+^ transient followed by oscillations. Notably, the incorporation of succinate at these conditions augmented the amplitude of these Ca^2+^ oscillations, indicating increased Ca^2+^ mobilization (Figure 4C).

Within the framework of neurohormonal amplification pathways, PKC activation is almost universally accepted as a major mechanism of GPCR-stimulated insulin secretion (55, 56). To discern the contribution of this family of serine-threonine kinases to SUCNR1-induced insulin secretion, we treated EndoC-βH5 cells with the pan-PKC inhibitor Gö 6983, and found that it partially blocked insulin release stimulated by succinate in high-glucose conditions (Figure 4D). Several G proteins are known to mediate GPCR signaling upon receptor activation (57). Given our finding that PKC signaling is implicated in SUCNR1-induced insulin secretion, we assessed the involvement of Gq, which is situated upstream of PKC (58). We used FR900359 to inhibit Gq activity in EndoC-βH5 cells, observing a complete blockade of succinate-induced potentiation of insulin release (Figure 4E). Overall, our data suggest that the succinate/SUCNR1 axis is a regulator of insulin secretion in a Gq- and PKC-dependent manner.

### Specific β cell ablation of SUCNR1 induces glucose intolerance due to β cell impairment.

**Figure 5:**
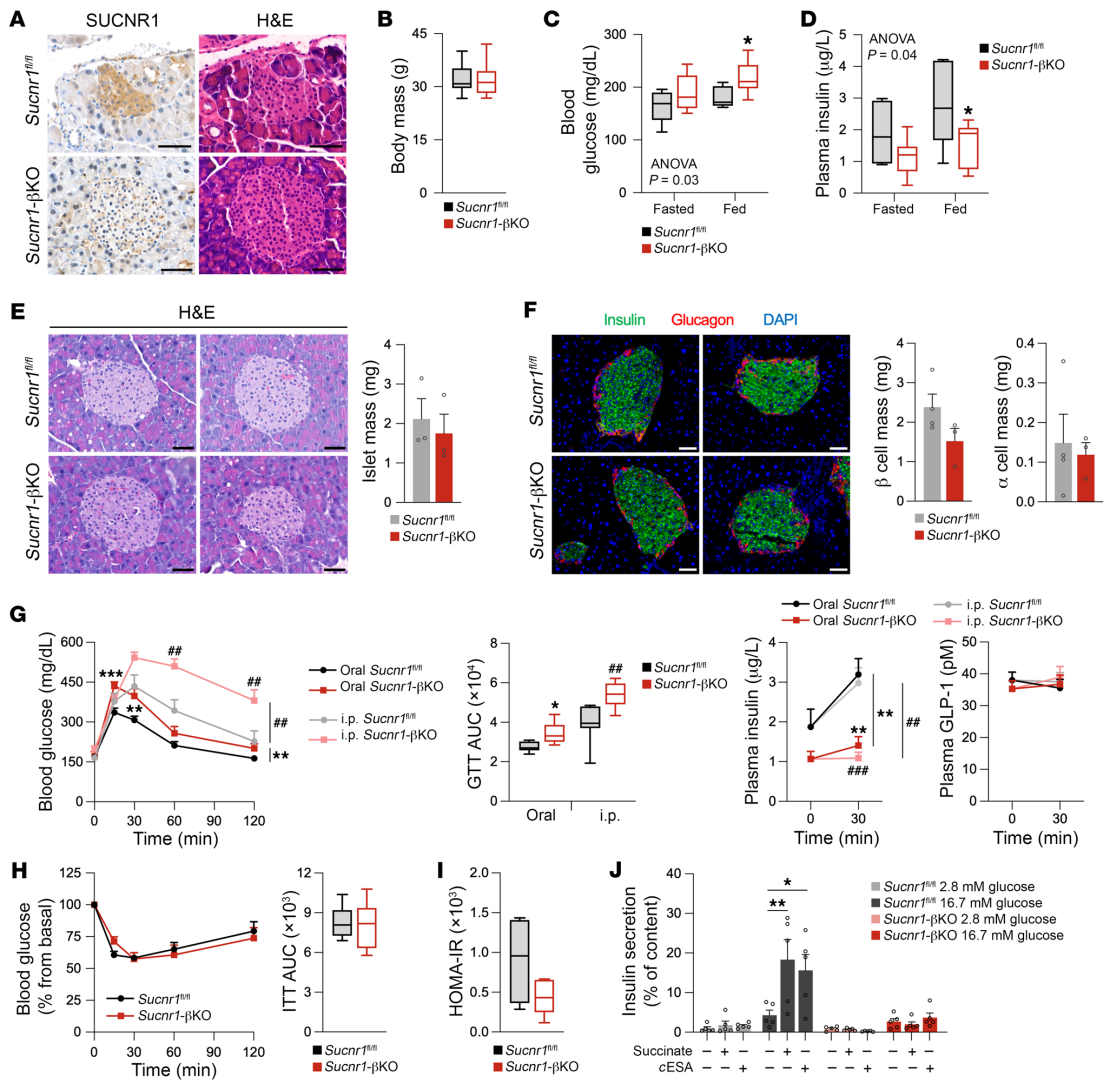
SUCNR1 in β cells is required for preserving insulin secretion and glucose homeostasis in HFD-fed male mice. (**A**) IHC staining of SUCNR1 in pancreas sections of control and *Sucnr1*-βKO mice accompanied by serial H&E staining. Scale bars: 50 μm. (**B**) Body mass of control and *Sucnr1*-βKO mice under HFD for 8 weeks (*n* = 8–9). (**C**) Blood glucose levels in control and *Sucnr1*-βKO mice in fasted or random-fed conditions (*n* = 8). (**D**) Plasma insulin levels in control and *Sucnr1*-βKO mice in fasted or random-fed conditions (*n* = 7–8). (**E**) Morphometric analysis of control and *Sucnr1*-βKO mice by H&E staining (*n* = 3). Scale bars: 50 μm. (**F**) Morphometric analysis of control and *Sucnr1*-βKO mice by immunofluorescence staining with insulin and glucagon and counterstaining with DAPI (*n* = 3–4). Scale bars: 50 μm. (**G**) Intraperitoneal (i.p.) and oral glucose tolerance tests in control and *Sucnr1*-βKO mice (*n* = 6–7). Displayed are the blood glucose levels, AUC, plasma insulin (*n* = 5–6), and GLP-1 levels (*n* = 5). (**H**) Insulin tolerance test in control and *Sucnr1*-βKO mice (*n* = 6–8). (**I**) HOMA-IR for control and *Sucnr1*-βKO mice (*n* = 5). (**J**) Insulin secretion in isolated islets from control and *Sucnr1*-βKO mice stimulated with or without 1 mM succinate or 100 μM *c*ESA at 2.8 or 16.7 mM glucose (*n* = 5 islet pools from 5–6 mice). Data are presented as mean ± SEM or as box-and-whisker plots indicating median, first and third quartiles, and maximum and minimum values. *P < 0.05, **P < 0.01, ***P < 0.001 vs. control mice, comparing experimental groups in orally administered mice, or in indicated pairwise comparisons; ^##^*P* < 0.01, ^###^*P* < 0.001 comparing experimental groups in i.p.-administered mice (Student’s *t* test in **B**, **E**, **F**, and **I** comparing 2 groups, or 2-way ANOVA with Bonferroni’s test for multiple comparisons in **C**, **D**, **G**, **H**, and **J**).

To further study the function of SUCNR1 in pancreatic β cells in vivo, we generated mice with β cell–specific *Sucnr1* deletion. We crossed mice carrying a conditional *Sucnr1* *loxP*-flanked locus (*Sucnr1^fl/fl^*) with knock-in heterozygous transgenic mice in which the Cre recombinase gene is introduced at the initiator codon of the *Ins1* endogenous gene locus (*Ins1^Cre/+^*). Accordingly, *Ins1^Cre/+^* mice do not show recombination in the brain and have glucose homeostasis and body weight gains similar to those of wild-type mice (59). The resulting offspring included both control mice (*Ins1^+/+^* *Sucnr1^fl/fl^*, referred to as *Sucnr1^fl/fl^* or control mice) and mice with β cell–specific *Sucnr1* ablation (*Ins1^Cre/+^* *Sucnr1^fl/fl^*, referred to as *Sucnr1*-βKO mice) (Supplemental Figure 1). These mice were born in a typical Mendelian fashion with no recognizable morphological differences between genotypes (data not shown). Immunohistochemical staining revealed a pronounced decrease in SUCNR1 content within pancreatic islets of *Sucnr1*-βKO mice (Figure 5A). No differences between genotypes were evident on a normal control diet (NCD) in terms of body weight, glucose and insulin tolerance, and insulin secretion at 16 and 54 weeks of age (Supplemental Figures 2 and 3).

We next challenged control and *Sucnr1*-βKO mice to an HFD to investigate whether a diet-induced metabolic stress phenotype existed. We found no differences in body weight between groups after 8 weeks of HFD feeding (Figure 5B). Remarkably, *Sucnr1*-βKO mice showed an overall higher diet-induced hyperglycemia than control mice, which was particularly significant in random-fed conditions (Figure 5C). Simultaneously, *Sucnr1*-βKO mice showed lower plasma insulin levels, which was also notably significant in random-fed conditions (Figure 5D). In a general morphometric examination of the pancreas using H&E staining, we observed a trend toward decreased islet mass in *Sucnr1*-βKO mice compared with control mice (Figure 5E). This pattern was also observed in the analysis of β and α cell mass by immunofluorescence in an additional cohort of mice (Figure 5F). Nonetheless, neither of these trends reached statistical significance.

To question whether specific β cell disruption of *Sucnr1* affected glucose response in an obesity context, we performed a glucose tolerance test (GTT) in mice fed an HFD. To consider potential differences in incretin-induced responses in β cells, we independently challenged mice to an intraperitoneal (i.p.) GTT and an oral glucose gavage GTT (OGTT). Results showed that the glucose response was higher in *Sucnr1*-βKO mice than in control mice for both tests, which was accompanied by a reduced insulin secretory response. These differences were not associated with changes in the glucose-associated GLP-1 response in *Sucnr1*-βKO mice on HFD (Figure 5G). Likewise, no appreciable changes were observed in insulin sensitivity measured directly by an insulin tolerance test (Figure 5H) or indirectly by assessment of the homeostasis model assessment index of insulin resistance (HOMA-IR) (Figure 5I).

To assess whether altered circulating insulin levels stemmed from impaired β cell function in vivo, we measured insulin secretion in isolated islets from control and *Sucnr1*-βKO mice. Under NCD conditions, islets from control mice demonstrated increased insulin secretion following succinate exposure in contrast to the secretion induced by high glucose alone, a phenomenon that was absent in islets from *Sucnr1*-βKO mice (Supplemental Figure 2E). In mice challenged with an HFD, islets from control mice showed enhanced insulin secretion upon SUCNR1 activation with succinate or *c*ESA during high-glucose conditions. Conversely, such a response was completely blunted in islets from *Sucnr1*-βKO mice (Figure 5J). Altogether, these data demonstrate that SUCNR1 plays an essential role in insulin secretion in vivo, particularly in an obesity context.

### Succinate functions as a hormone and is associated with the potentiation of insulin secretion in humans.

**Figure 6:**
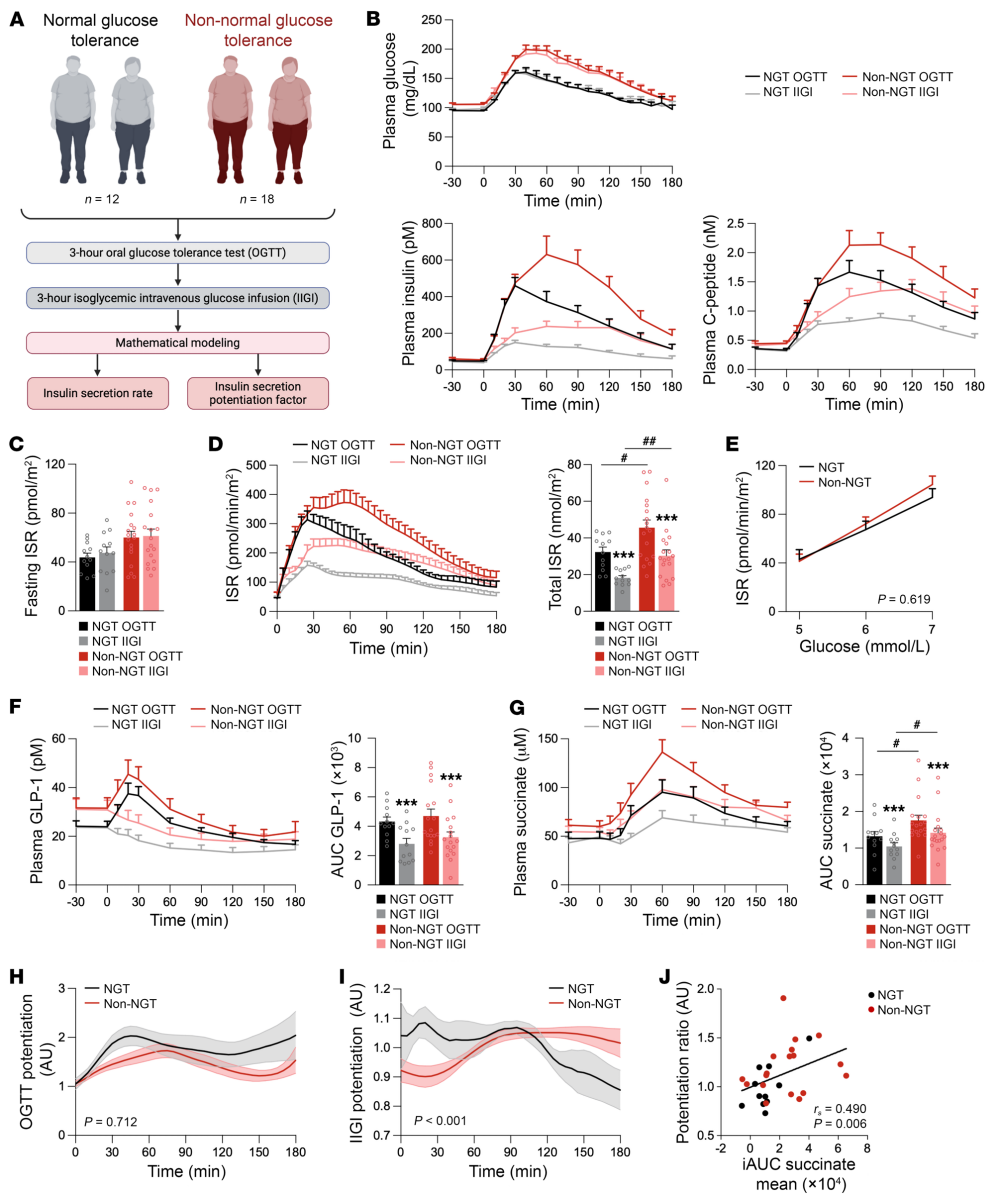
Succinate response is heightened in patients without NGT and is associated with potentiation of insulin secretion. (**A**) Study cohort and study schematic. (**B**) Plasma glucose, insulin, and C-peptide levels during the oral glucose tolerance test (OGTT) and isoglycemic intravenous glucose infusion (IIGI) in patients with NGT (*n* = 12) and those without NGT (non-NGT; *n* = 18). (**C**) Fasting insulin secretion rate (ISR) in NGT and non-NGT groups. (**D**) ISR during OGTT and IIGI tests in NGT and non-NGT groups. (**E**) β Cell glucose sensitivity represented as a dose-response function between ISR and glucose concentrations for both groups. (**F**) Plasma GLP-1 levels during OGTT and IIGI for both groups. (**G**) Plasma succinate levels during OGTT and IIGI for both groups. (**H**) Incretin-related potentiation calculated during OGTT in relation to IIGI for both groups. (**I**) Potentiation calculated during IIGI for both groups. (**J**) Correlation between the potentiation factor mean and the AUC of succinate during IIGI for both groups (*n* = 30). Data are presented as mean ± SEM. ***P < 0.001 comparing OGTT and IIGI; ^#^*P* < 0.05, ^##^*P* < 0.01 comparing NGT and non-NGT individuals (paired and unpaired Student’s *t* tests in **C**, **D**, **F**, and **G**, Mann-Whitney *U* test in **D**, **F**, and **G**, Wilcoxon’s test in **D**, **F**, and **G**, 2-way ANOVA tests in **E**–**I**, or Spearman’s rank correlation coefficient in **J**).

To explore the physiological significance of succinate as an insulin secretagogue, we investigated the potential link between circulating succinate kinetics and pancreatic β cell function in response to an OGTT and to an isoglycemic intravenous glucose infusion (IIGI) in a cohort of individuals with normal glucose tolerance (NGT) or individuals without NGT according to the American Diabetes Association criteria (60) (Figure 6A; clinical and anthropometric data in Table 1). As expected, individuals in the non-NGT group had higher fasting plasma glucose and 2-hour glucose during the OGTT and were insulin resistant. Accordingly, these patients had higher glucose excursions during both OGTT and IIGI, which were accompanied by higher insulin and C-peptide responses (Figure 6B). Analysis of the insulin secretion rate (ISR) revealed no significant differences between groups during fasting, although an upward trend was observed in the non-NGT group (Figure 6C). Conversely, stimulated ISR was higher in the non-NGT group during both OGTT and IIGI (Figure 6D), which agrees with relative hyperglycemia and the characteristic hyperinsulinemia of insulin-resistant states. These differences were not associated with variations in the estimated β cell glucose sensitivity (Figure 6E). Notably, there was a tendency toward elevated fasting levels of ciraculating succinate in the non-NGT group when compared with the NGT group (Table 1). However, it is important to consider that the control group includes individuals with varying degrees of overweight. This aspect may contribute to the observed trend not reaching statistical significance, potentially masking the differences previously reported in individuals with obesity (61, 62). No differences were found between groups in terms of the GLP-1 response (a recognized factor of the amplifying pathway) to the glucose challenge, independently of the route of glucose administration (Figure 6F), or the total incretin effect (Table 1). Strikingly, however, the succinate response to glucose, which was found to be higher after nutrient passage through the gut (similar to GLP-1), was significantly higher in the non-NGT group than in the NGT group (Figure 6G).

According to the GLP-1 response (Figure 6F), incretin potentiation was similar between groups during the OGTT (*P* = 0.7118) (Figure 6H). Contrastingly, during the IIGI, in which the incretin effect is considered negligible, the curve of glucose-induced potentiation exhibited a modest decrease in individuals with NGT, while conversely, there was a slight increase in individuals without NGT over time (*P* < 0.0001) (Figure 6I). To determine whether succinate is associated with insulin secretion potentiation, we performed a correlation analysis between the potentiation ratio and the succinate response during the IIGI. A significant correlation between both variables was observed (Figure 6J). Overall, this analysis indicates that succinate is associated with insulin secretion in humans, which may be particularly relevant to the hyperinsulinemia developed in insulin-resistant states.

## Discussion

Our study uncovers what we consider to be a hitherto unknown molecular link between the succinate/SUCNR1 axis and β cell function. While previous research in this area primarily focused on the ability of cell-permeable succinate analogs to stimulate insulin secretion through TCA cycle–anaplerosis (13–16), our findings reveal that beyond its intracellular functions, extracellular succinate stimulates insulin secretion in hyperglycemic states in mice and humans via SUCNR1 activation. This mechanism represents a significant amplifying pathway for insulin secretion, which may be relevant in prediabetic states in which the oversecretion of insulin is an adaptive response to overcome insulin resistance.

Several GPCR-mediated mechanisms cooperate in β cells to regulate insulin secretion (63). Our study establishes that SUCNR1 in β cells potentiates insulin secretion in response to high glucose levels, similarly to incretins such as GLP-1 and GIP (64). Notably, in humans, we also observed this stimulatory effect on insulin secretion under non-glucose conditions, consistent with previous findings indicating species-specific variations in insulin secretion mechanisms to meet specific physiological needs (65, 66). With regard to the transduction pathways underlying this insulinotropic effect, SUCNR1 signaling differs among cell types (53), but with a preference to engage Gi or Gq proteins (36, 67). We identified a Gq- and PKC-dependent mechanism linking SUCNR1 activation to the potentiation of insulin secretion. Some studies have speculated that increases in [Ca^2+^]_i_ observed upon SUCNR1 activation (which we also observed in β cells) are mediated by Gi and βγ subunit–induced PLC-β activation rather than Gq (68, 69), but recent studies have established that Gq engagement is required for PKC- and [Ca^2+^]_i_-mediated responses upon SUCNR1 activation in immune cells (36, 70). Likewise, other GPCRs, such as GPR40 (activated by free fatty acids), have been reported to use pathways mediated by Gq, PKC, and [Ca^2+^]_i_, which are known to trigger insulin secretion (71, 72).

Our findings identify the succinate/SUCNR1 axis as an amplifying route for insulin secretion in response to hyperglycemia. Indeed, high glucose significantly increases β cell sensitivity to extracellular succinate by elevating SUCNR1 expression. Accordingly, SUCNR1 expression is significantly greater in islets from donors with obesity and T2D than in lean control donors, which fits with our previous findings of higher circulating succinate levels in the former (61, 62). The upregulation of SUCNR1 associated with hyperglycemia differs, however, from the downregulation reported for GLP-1R in conditions associated with high glucose (73, 74). Notably, several identified risk variants for T2D have been mapped at the *SUCNR1* locus or are in close proximity to *cis*-regulatory elements (40, 42, 75). Collectively, these findings might suggest that modifications in SUCNR1 expression in β cells in some diseases are linked to metabolic cellular adaptations and an elevated risk of developing T2D.

SUCNR1 activation is traditionally recognized as a mechanism bolstering tissue damage (76–81). Indeed, chronic elevation of extracellular succinate is found in low-grade inflammatory metabolic disorders such as cardiovascular disease, obesity, and T2D (26, 61, 82, 83). However, circulating succinate levels also rise transiently in response to normal physiological processes such as exercise (12) and food intake (33). The nutritional regulation of succinate is dependent on glucose sensing by the intestine and the metabolic status of the subject (33), suggesting a key signaling role for succinate in metabolic processes related to energy management (10). Along this line, we recently showed that the succinate/SUCNR1 axis is a regulator of leptin production in adipocytes (84). This study, in addition to our current findings identifying a function for succinate as an insulin secretagogue, strongly supports a role for SUCNR1 as part of the metabolite-sensing machinery allowing cells to coordinate energy homeostasis. Consistent with this hypothesis, we show that β cell–specific depletion of SUCNR1 in mice leads to an aberrant metabolic phenotype characterized by increased glucose intolerance and defects in insulin secretion under HFD feeding. While there is a discernible trend toward reduced β cell mass in *Sucnr1*-βKO mice under HFD conditions, suggesting a potential impact of SUCNR1 on β cell proliferation, it is important to note that this change lacks statistical significance. As a result, we propose that SUCNR1 exerts its primary influence on the functionality of adult β cells, rather than on their development or the typical compensatory alterations in β cell mass observed during obesity (85). The observed similarities in the GLP-1 response between *Sucnr1*-βKO mice and control mice on an HFD, coupled with the pronounced impairment in insulin secretion and glucose tolerance in the *Sucnr1*-βKO mice, underscore the pivotal role of SUCNR1 in preserving insulin secretion. Despite the absence of incretin response both in *Sucnr1*-βKO mice and control mice in diet-induced obesity, β cells in *Sucnr1*-βKO mice fall short in counteracting hyperglycemia, which emphasizes the critical involvement of the succinate/SUCNR1 axis in this scenario. Moreover, our findings align with an earlier study using global-*Sucnr1*-knockout mice, which exhibited progressive glucose intolerance under HFD and an impaired insulin response, occurring as part of a complex phenotype (25). However, other organs (e.g., adipose tissue) also influence these phenotypes, underscoring the importance of analyzing the effects of SUCNR1 in specific cell types (10, 24, 84).

Our study in patients highlights the significance of the succinate/SUCNR1 axis in preserving glucose homeostasis during metabolic challenges. Prediabetes, a state observed in patients without NGT, is characterized by glucose intolerance resulting from systemic insulin resistance, which is substantially compensated for by the oversecretion of insulin (86) through mechanisms that remain elusive. When individuals were challenged with glucose, we observed a transient increase in succinate levels following glucose administration, particularly elevated in prediabetic individuals, while the GLP-1 response mirrored that of individuals with NGT. This suggests that factors beyond incretins may play a role in modulating the enhanced insulin secretion seen in prediabetic conditions. In this context, succinate emerges as a regulator of the observed hyperinsulinemic response in prediabetic patients. No significant differences in incretin-related potentiation were observed between the groups during an OGTT, likely because of preserved operation of the incretin system in patients without NGT. However, during intravenous glucose infusion, where the incretin effect on insulin secretion is negligible (87), we found a positive correlation between the succinate response and insulin secretion potentiation. The succinate response to glucose is higher during — but not exclusive to — the oral challenge (33), which prompts the consideration of other succinate sources in addition to the intestine. We observed that β cells secrete more succinate when they are exposed to high glucose, which is consistent with previous studies showing that hyperglycemia can also lead to local increases in succinate (80, 88), likely due to overactivation of the TCA cycle (89). Thus, it is tempting to speculate that succinate endogenously produced in β cells could be partly responsible for SUCNR1 engagement in an autocrine fashion. Succinate derived from the gut microbiota is also a potential source (10), and indeed, other metabolites derived from the gut microbiota, including short-chain fatty acids, have been described as insulin secretagogue GPCR ligands (90, 91).

While the human study offers valuable associative insights, the concurrent presence of heightened glucose intolerance, insulin secretion, and succinate response in prediabetic conditions prevents us from conclusively establishing a direct link between the succinate/SUCNR1 axis and insulin secretion. However, our integrated findings encompassing patient observations and preclinical models collectively suggest that the succinate/SUCNR1 axis in β cells intricately regulates insulin secretion and helps maintain glucose homeostasis, particularly under metabolic stress conditions. The observed upregulation of SUCNR1 in human islets during obesity and diabetes, coupled with evidence from our β cell SUCNR1 knockout model, points to a compensatory mechanism aimed at counteracting metabolic challenges.

Our findings highlight SUCNR1 as a potential therapeutic target to enhance insulin secretion in the context of T2D management. Indeed, the nutritional modulation of succinate is lost in patients with morbid obesity and T2D (33). Nonetheless, further research is needed given the broad expression of SUCNR1 and its pleiotropic functions (10). While the succinate/SUCNR1 axis is crucial for the resolution of inflammation (24, 36), activation of SUCNR1 in an inflammatory context could be detrimental owing to its proinflammatory effects in the acute phase of the immune response (78, 92). Along these lines, decreasing the higher levels of circulating succinate acting on its intestinal source, rather than directly activating SUCNR1, has recently emerged as a potential strategy for treating metabolic diseases (93). Whether this strategy could also be effective in recovering the function of SUCNR1 in insulin secretion deserves further analysis.

## Methods

Further information is available in Supplemental Methods.

### Sex as a biological variable.

In human studies, we included both male and female individuals. Sex was consistently considered as a variable, yet no differences were detected. In animal studies, we exclusively examined male animals. The relevance of our findings to female animals is unknown, though we observed no sex-dependent differences in humans.

### Human cohort.

The study was carried out at the Unit of Functional Studies, Institut d’Investigació Sanitària Pere Virgili, Hospital Universitari Joan XXIII, Tarragona, Spain. As part of a screening protocol for obesity in the general population, healthy asymptomatic individuals were selected from those who visited the Endocrinology Service. The main inclusion criteria were as follows: age between 18 and 65 years, BMI less than 35 kg/m^2^, absence of underlying pathologies (cancer, liver disease, kidney disease, or systemic inflammation) on physical examination or diagnostic tests other than those associated with an excess of weight, and signing of the protocol/informed consent. Additionally, inclusion criteria required a level of hemoglobin A_1c_ less than 6.5% and a fasting glucose level less than 126 mg/dL on 2 separate occasions. After the initial triage, 30 individuals were selected to be submitted to an oral glucose tolerance test (OGTT) with 75 g of glucose. Individuals were classified according to the American Diabetes Association (ADA) criteria (60) as having normal glucose tolerance (NGT; fasting glucose levels ≤ 110 mg/dL and a normal OGTT response) or non-normal glucose tolerance (non-NGT; fasting glucose levels ≥ 110 and < 126 mg/dL, or impaired glucose tolerance or diabetes during an OGTT following the ADA criteria) (clinical information summarized in Table 1). The main exclusion criteria were pregnancy and lactation; vegetarianism, veganism, or eating disorders; chronic treatment with antiinflammatory agents, antibiotics, or cortisol; and major psychiatric antecedents, alcoholism, or drug abuse.

### Assessment of β cell function and succinate dynamics in humans.

All participants underwent 2 procedures on separate occasions. We first applied a 3-hour OGTT and subsequently applied a 3-hour isoglycemic intravenous glucose infusion (IIGI), reproducing the plasma glucose time curve recorded during the OGTT using an ad hoc algorithm. Plasma glucose was determined every 10 minutes during both procedures, whereas insulin, C-peptide, GLP-1, and succinate were determined in plasma samples at –30, 0, 10, 20, 30, 60, 90, 120, 150, and 180 minutes. Body composition was determined by electric bioimpedance (Tanita Europe BV). During blood collection, a specific DPP-IV inhibitor was present in the tubes (DPP4-010, Merck KGaA). Blood samples were always kept on ice and stored at –8°C. Plasma lipid, hepatic, and renal profiles were determined by standard enzymatic methods. Plasma glucose was determined by the glucose oxidase method (GM-9, Analox). Plasma insulin and C-peptide levels were determined by an immunochemiluminometric assay (ADVIA Centaur, Siemens Healthcare). Total plasma GLP-1 levels (7–36 and 9–36) were determined by ELISA (EZGLP1T-36 K, Merck). Plasma succinate was determined in plasma filtrates (10 kDa) using a fluorometric assay (EnzyChrom Succinate Assay Kit, BioAssay Systems).

The areas under the curve (AUCs) were calculated using the trapezoid rule. Insulin sensitivity was estimated from the plasma glucose and insulin responses to oral glucose using the OGTT-derived index of insulin sensitivity (OGIS) (94). In vivo insulin secretion, β cell function parameters, and the incretin effect were calculated by simultaneous modeling analysis of the IIGI and the OGTT, as previously described (95). The model reconstructs insulin secretion from plasma C-peptide using an established model of C-peptide kinetics (96), and it extends a widely used model for the analysis of the OGTT (97). In brief, the model describes the relationship between insulin secretion and glucose concentration during the IIGI as a quasilinear function (i.e., the dose-response). This response is modulated during the test by a time-dependent factor, which averages to 1. This time-dependent factor, denoted as glucose-induced potentiation, accounts for potentiation phenomena that typically prompt glucose-dependent insulin secretion higher at the end of the test compared with the beginning. The model also includes a term describing early secretion, related to first-phase insulin release. Insulin secretion during the OGTT is described by the same terms, although the dose-response is multiplied by another time-dependent factor, typically greater than 1, which accounts for the effect of incretin hormones on insulin secretion. This time-dependent factor is denoted incretin potentiation, and its AUC is a measure of the total incretin effect. To study a potential relationship between the insulin secretion potentiation during the IIGI and succinate dynamics, we used the potentiation ratio between 2 hours and the baseline, and the corresponding 2-hour incremental succinate AUC mean.

### Animals.

Male Wistar rats (Charles River Laboratories) were housed in the Animal Facility of Miguel Hernández University. All mice were housed in the Animal Facility of the Faculty of Medicine and Health Sciences, Universitat Rovira i Virgili. Housing conditions consisted of automated 12-hour light/12-hour dark cycles at 20°C–22°C, 40%–60% relative humidity, and ad libitum access to NCD (3.1 kcal% fat; SAFE Diets, A04) or HFD (60 kcal% fat; Research Diets Inc., D12492). HFD availability to mice started at 8 weeks of age and continued until the study endpoint. Male mice were weighed before any experimental procedure. At the end of the study, mice were fasted for 16 hours and then euthanized by cervical dislocation. Tissues were collected and immediately snap-frozen in liquid N_2_ or fixed in 4% formaldehyde.

Male wild-type C57BL/6 mice and male diabetic *db/db* mice were obtained from Charles River Laboratories. β Cell–specific *Sucnr1*-knockout mice (*Sucnr1^fl/fl^* *Ins1^Cre/+^*, *Sucnr1*-βKO) were obtained in the F_2_ generation by crossing of parental homozygous *Sucnr1*-floxed mice (*Sucnr1^fl/fl^* *Ins1^+/+^*, control) (24) and *Ins1^Cre/+^* mice, the latter provided by J. Ferrer (Centre for Genomic Regulation, Barcelona, Spain). Briefly, *Ins1^Cre/+^* mice were generated by a knock-in strategy into one endogenous *Ins1* allele and present more specificity for restricted β cell Cre expression, avoiding recombination in other tissues, including the brain (59). Control Cre-negative mice were generated in the same litters along with *Sucnr1*-βKO mice (Supplemental Figure 1). All resulting mice were generated on a pure C57BL/6 background and genotyped by PCR using specific primers to amplify *Sucnr1^+^* and *Sucnr1^fl^* alleles (300 bp product for the wild-type allele and 450 bp product for the floxed form) and to amplify *Ins1^+^* or *Ins1^Cre^* alleles (524 bp for the wild-type and 865 bp for the mutated structure).

### Physiological experiments on mice.

For the i.p. and oral GTT, 16- and 54-week-old male mice were fasted for 16 hours, and blood glucose was measured before and 15, 30, 60, and 120 minutes after administration of glucose (2 g/kg body weight). For the ITT, male mice were fasted for 4 hours, and blood glucose was measured before and 15, 30, 60, and 120 minutes after an i.p. injection of recombinant human insulin (0.75 IU/kg body weight; Actrapid, Novo Nordisk). Blood glucose was measured using a glucose meter (Accu-Chek, Roche). The AUC for glucose was calculated using the trapezoidal rule for any tolerance test (98). Plasma insulin was determined using a Mouse Insulin Ultrasensitive ELISA kit (Mercodia). GLP-1 levels were analyzed in plasma using a Mouse GLP-1 ELISA kit (Crystal Chem).

### Cell lines.

EndoC-βH1 is an engineered cell line obtained from a male human pancreas subjected to lentiviral transfection of the SV40 large T antigen (SV40LT) expressed under the control of the insulin promoter and the human telomerase reverse transcriptase (hTERT) (99). These cells present several similarities to primary human β cells (100). EndoC-βH1 cells were cultured as previously described (101). Briefly, before seeding of the cells, the culture dishes and plates were coated with a mixture of DMEM (4.5 g/L glucose; Thermo Fisher Scientific), fibronectin (2 μg/mL; Thermo Fisher Scientific), and extracellular matrix (1%; Sigma-Aldrich). The coated flasks were then incubated for at least 1 hour in a 5% CO_2_ atmosphere at 37°C. EndoC-βH1 cells were maintained at 37°C and 5% CO_2_ in DMEM-based medium consisting of 1 g/L glucose, 2% BSA, 50 μM 2-mercaptoethanol (Sigma-Aldrich), 10 mM nicotinamide (Sigma-Aldrich), 5.5 μg/mL transferrin (Sigma-Aldrich), 6.7 ng/mL sodium selenite (Sigma-Aldrich), and 1% penicillin-streptomycin (Thermo Fisher Scientific). To measure SUCNR1 protein levels, cells were starved for 16 hours in 2.8 mM glucose DMEM-based medium followed by treatment with 5.5, 11.1, 16.7, or 25 mM glucose for 24 hours.

The latest iteration of the human EndoC cell series is the EndoC-βH5 cell line, which was purchased from Human Cell Design. These cells are derived from human female fetal pancreatic tissue and demonstrate an improved insulin secretion response, heightened sensitivity to incretins, and more pronounced expression of β cell identity markers in comparison with other human β cell lines, displaying a greater similarity to primary adult β cells. The generation process of these cells was similar to previous models, but the SV40LT, hTERT, and herpes simplex virus-1 thymidine kinase transgenes were removed through Cre-mediated recombination to produce mature, non-proliferative cells (51). EndoC-βH5 cells were seeded at 1 × 10^5^ cells per well in 96-well Cell^+^ plates (Sarstedt) pretreated with βCOAT matrix (Human Cell Design), and cultured for 10 days before performing of the GSIS assays. Cells were maintained in ULTI-β1 medium (Human Cell Design), and the medium was changed 4 hours after seeding, when all viable cells had attached, and then again 4 days later.

MIN6 is an insulinoma-derived cell line from transgenic male mice transfected with SV40 T antigen, with the ability to secrete insulin in the presence of glucose. MIN6 cells were maintained under standard culture conditions (102). Briefly, MIN6 cells were cultured at 37°C and 5% CO_2_ in DMEM consisting of 4.5 g/L d-glucose, 2 mM l-glutamine (Thermo Fisher Scientific), 15% fetal bovine serum (Thermo Fisher Scientific), 1% penicillin-streptomycin, 20 mM HEPES (Thermo Fisher Scientific), and 50 μM 2-mercaptoethanol. To assess *Sucnr1* gene expression levels, cells were starved for 16 hours in 2.8 mM glucose DMEM-based medium followed by stimulation with 5.5, 11.1, or 16.7 mM glucose for 3 and 24 hours.

### Gene expression analysis.

RNA was extracted from tissues or cells using TRIzol Reagent, and cDNA was synthesized using the High-Capacity cDNA Reverse Transcription kit (both from Thermo Fisher Scientific). Quantitative real-time PCR amplification was performed on a 7900HT Fast Real-Time PCR System with the TaqMan Gene Expression Assay from Thermo Fisher Scientific (probes are listed in Supplemental Table 3). Relative mRNA levels were calculated using the comparative 2^–ΔΔCt^ method, corrected for the corresponding housekeeping gene expression levels, and displayed as log_2_ for normalization.

### Western blotting.

Cells or tissues were lysed in M-PER buffer containing Halt Protease and Phosphatase Inhibitor Cocktails, and protein concentration was measured using the BCA protein assay kit (all from Thermo Fisher Scientific). Samples were resolved on SDS-PAGE gels, and proteins were transferred onto PVDF membranes (Merck KGaA). Membranes were blocked in a blocking buffer consisting of 5% blotting-grade blocker nonfat dry milk (Bio-Rad) and incubated with primary antibodies overnight at 4°C. A polyclonal primary antibody against SUCNR1 (NBP1-0086) was obtained from Novus Biologicals. A monoclonal anti–β-actin antibody was purchased from Sigma-Aldrich (A1978). Primary antibodies against phospho-PKCδ (T505; 9374), phospho-PKCζ/λ (T410/403; 9378), phospho-Akt (S473; 4058), phospho–GSK-3α/β (S21/9; 9331), phospho-ERK1/2 (T202/Y204; 9101), phospho-p38 (T180/Y182; 4511), and phospho-CREB (S133; 9198) were obtained from Cell Signaling Technology. Membranes were incubated with the respective HRP-linked secondary antibodies for 1 hour and developed using the Pierce ECL Western Blotting Substrate or SuperSignal West Femto Maximum Sensitivity Substrate (Thermo Fisher Scientific). Band intensity was captured using the iBright CL1000 Imaging System, and analysis was performed with iBright Analysis Software (Thermo Fisher Scientific).

### Glucose-stimulated insulin secretion assays.

MIN6 cells were washed twice in Krebs-Ringer-Phosphate-HEPES (KRPH) buffer (5 mM Na_2_HPO_4_, 1 mM MgSO_4_, 1 mM CaCl_2_, 136 mM NaCl, 4.7 mM KCl, 20 mM HEPES [pH 7.4], and 0.5% BSA), incubated in KRPH buffer with 2.8 mM d-glucose for 1 hour at 37°C, 5% CO_2_, and saturated humidity, and then washed twice with KRPH buffer. Cells were then incubated for 1 hour in KRPH buffer containing either 2.8 or 16.7 mM d-glucose and the corresponding stimulus. EndoC-βH5 cells were subjected to GSIS assays following the manufacturer’s protocol (Human Cell Design). In brief, cells were starved in ULTI-ST medium for 24 hours, then washed twice in βKrebs medium (both from Human Cell Design) supplemented with 0.1% BSA (βKrebs-BSA) and incubated in βKrebs-BSA medium for 1 hour at 37°C, 5% CO_2_, and saturated humidity. Cells were then stimulated for 40 minutes with 0 or 20 mM glucose and the indicated stimulus in βKrebs-BSA medium. At the end of GSIS assays, the conditioned medium (CM) from cells was collected and centrifuged at 300 RCF for 15 minutes at 4°C, and the supernatant was stored at –80°C. The remaining cells or islets were washed once in PBS and lysed with M-PER buffer with Halt Protease Cocktail (Thermo Fisher Scientific) for protein quantification.

Pools of 300 islets (100 islets per mouse) were preincubated with Krebs-Ringer-Bicarbonate-HEPES (KRBH) buffer, consisting of 115 mM NaCl, 2.5 mM CaCl_2_·2H_2_O, 24 mM NaHCO_3_, 5 mM KCl, 1 mM MgCl_2_·6H_2_O, 20 mM HEPES, and 0.5% BSA, pH 7.4, and containing 2.8 mM glucose at 37°C and 5% CO_2_, for 2 hours. Subsequently, sets of 10 islets per condition, in quintuplicates, were incubated in KRBH buffer with either 2.8 mM or 16.7 mM glucose, with or without the addition of the indicated stimuli. After 1 hour, the CM from islets was collected and centrifuged at 300*g* for 15 minutes at 4°C, and the supernatant was then stored at –80°C. Total insulin contents were extracted using a lysis buffer composed of 5.75% acetic acid (Sigma-Aldrich) and 0.1% BSA.

Reagents for GSIS assays were 0.5–1 mM disodium succinate (W327700, Sigma-Aldrich), 50–100 μM *cis*-epoxysuccinic acid (*c*ESA; E0449, TCI Chemicals), 1 μM NF-56-EJ40 (HY-130246, MedChemExpress), 1 μM Gö 6983 (2285, Tocris), and 1 μM FR900359 (33666, Cayman Chemical Co.). Inhibitors were preincubated for 3 hours in all media preparations during the assays at the reported concentrations. The insulin content in the CM and lysates was measured using Mouse or Human Insulin ELISA kits (Mercodia).

### Measurement of cytosolic Ca^2+^.

Intracellular free Ca^2+^ was determined in MIN6 cells grown on glass coverslips (Menzel-Gläser). At 80% confluence, the coverslip was carefully transferred to a Petri dish containing 3 mL of Locke-HEPES buffer (LH) comprising 120 mM NaCl, 10 mM KCl, 15 mM NaHCO_3_, 3.3 mM MgCl_2_, 2.6 mM CaCl_2_, 2.8 mM d-glucose, and 10 mM HEPES, pH 7.4, and including 6 μM fura-2-acetoxymethyl ester (Fura-2 AM; Thermo Fisher Scientific). Cells were incubated at 37°C for 30 minutes in a cell incubator. For fluorescence recordings, the coverslip was carefully rinsed in LH, mounted in a specific holder (coverslip accessory L2250008, PerkinElmer), and placed in a quartz cuvette containing 1.3 mL of LH. Measurements were made at 37°C with continuous mild stirring in an LS50B PerkinElmer fluorescence spectrometer equipped with a fast-filter accessory for Fura-2 AM fluorescence ratio measurements. Emission data (510 nm) were collected with alternate excitation at 340 and 380 nm, and the ratio F_340_/F_380_ was calculated in real time using proprietary software (FL WinLab 2.0, PerkinElmer). The mobilization of cytosolic Ca^2+^ by extracellular succinate was evaluated before and after the increase of glucose to 16.7 mM.

Ca^2+^ measurements were also performed in isolated islets from 12-week-old male mice. After isolation, islets were allowed to recover for 2 hours at 37°C and then incubated with 2 μM Fura-2 AM at room temperature. Then, islets were transferred to an imaging chamber mounted on a Zeiss Axiovert 200 inverted microscope equipped with a ×40 objective. During experiments, islets were continuously perifused with a KRBH buffer (pH 7.35) containing the corresponding stimuli. Images were acquired using a Hamamatsu C9100 digital camera (Hamamatsu Photonics). Intracellular Ca^2+^ levels were represented as the ratio F_340_/F_380_. The AUC at 8 mM glucose was calculated for 5 minutes before the application of succinate. The AUC of succinate was also calculated for 5 minutes, but 10 minutes after succinate application to allow its equilibration in the perifusion chamber.

### Statistics.

The data presented in tables and graphics are displayed as either the mean ± SD or mean ± SEM, as indicated. In the box-and-whisker plots, the median is indicated by a center line, while the upper and lower quartiles are represented by the box limits, and the minimum to maximum range of the data set is indicated by the whiskers. The Shapiro-Wilk normality test was applied before any contrast hypothesis testing. When the data were normally distributed, a 2-tailed Student’s *t* test was used to compare differences between 2 groups, applying Welch’s correction if a difference between the variations of the compared groups was observed. For comparisons involving multiple groups, a 1-way ANOVA was performed; Dunnett’s post hoc test was used for comparing multiple groups with a control group, while Tukey’s post hoc test was applied for comparing all groups with each other, to correct for multiple comparisons. For time course experiments, a 2-way ANOVA with Bonferroni’s correction for multiple comparisons was conducted. In cases in which the data did not follow a normal distribution, the non-parametric Mann-Whitney 2-tailed *U* test for unmatched pairs, Wilcoxon’s signed-rank test for matched pairs, or the Kruskal-Wallis test with Dunn’s test for multiple comparisons was used to compare differences as appropriate. Pearson’s or Spearman’s correlations were employed to calculate linear associations between continuous variables. Both GraphPad Prism 9 and RStudio software for Mac OS and IBM SPSS 27 software for Windows OS were used for the statistical analyses, and a *P* value of less than 0.05 was considered statistically significant.

### Study approval.

The conduct of the study protocols was in accordance with the ethical principles outlined in the Declaration of Helsinki and received authorization from the relevant local ethics committees (Institut d’Investigació Sanitària Pere Virgili Ethics Research Committee reference 204/2017). Prior to their participation, all individuals were given a thorough explanation of the study protocol and provided written informed consent.

The use of human islets for gene expression analysis was approved by the local Ethics Committee of Hospital Universitari de Bellvitge (approval PR239/13), and signed consent was obtained from donor relatives. All studies and protocols involved in human islet procurement and experimentation (for the protein expression analysis) were approved by the Joslin Diabetes Center’s Committee on Human Studies (approval CHS#5-05).

All animal studies conformed to the ARRIVE guidelines and were carried out under the supervision and approval of the Animal Welfare and Governmental Ethics Committee of the Universitat Rovira i Virgili (project reference 10970). All experimental procedures involving animals conformed to the European Union Directive 2010/63/EU and the European Commission Recommendation 2007/526/EC on the protection of animals used for experimental and other scientific purposes, enacted under Spanish Royal Decrees 53/2013 and 118/2021.

### Data availability.

All data points presented in the graphs are detailed in the Supporting Data Values file.

## Author contributions

Conceptualization was the responsibility of JSB, BA, JV, and SFV. Methodology was the responsibility of JSB, BA, CC, ME, JB, MRDD, IQ, DFDJ, L Marroquí, RB, EM, and FXS. Formal analysis was conducted by JSB, BA, JB, FXS, AT, and AM. Investigation was conducted by JSB, BA, CC, ME, MRDD, IQ, JB, CNR, MMRP, L Marroquí, DFDJ, L Martínez, and RB. Resources were provided by L Marroquí, AT, AM, EM, RNK, JV, and SFV. Original draft writing was conducted by JSB. Manuscript review and editing were the responsibility of JSB, AT, AM, RNK, JV, and SFV. Supervision was carried out by JV and SFV. Funding was acquired by JV and SFV.

## Supplementary Material

Supplemental data

Unedited blot and gel images

Supporting data values

## Figures and Tables

**Table 1:**
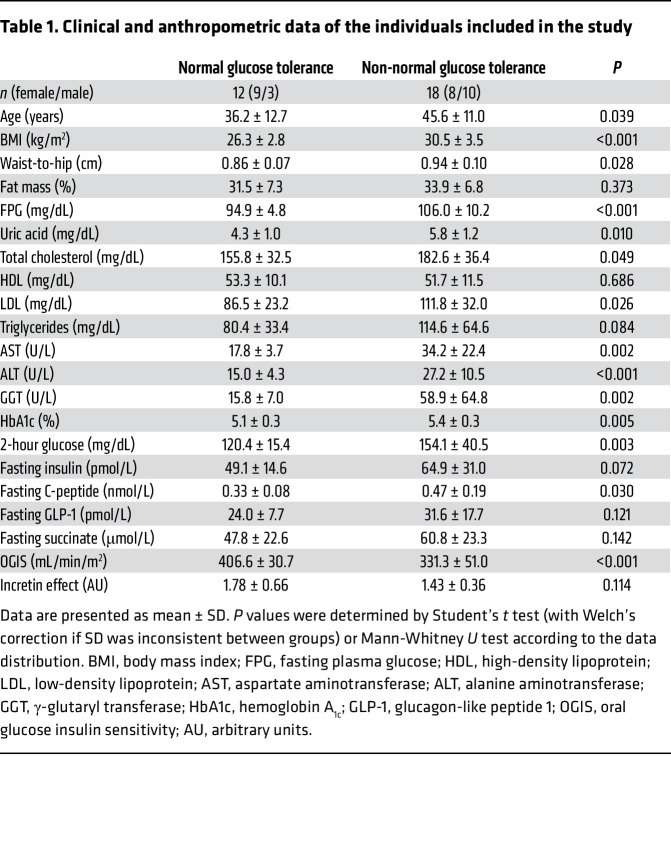
Clinical and anthropometric data of the individuals included in the study

## Version 1 05/07/2024

In-Press Preview

## Version 2 06/17/2024

Electronic publication
