# Supplementary material for: SUCNR1 regulates insulin secretion and glucose elevates the succinate response in people with prediabetes
Source: J Clin Invest. 2024 May 7;134(12):e173214. doi: 10.1172/JCI173214 (PMC11178533; doi:10.1172/JCI173214)
Supplement: Supplemental data [file jci-134-173214-s142.pdf]

Supplemental Data

SUCNR1 Regulates Insulin Secretion and Glucose Elevates the Succinate Response in People with Prediabetes

Joan Sabadell-Basallote[1-4], Brenno Astiarraga[1,2], Carlos Castaño[1,2], Miriam Ejarque[1,2], Maria Repollés-de-Dalmau[1-3], Ivan Quesada[2,5], Jordi Blanco[3], Catalina Núñez-Roa[1,2], M-Mar Rodríguez-Peña[1,2], Laia Martínez[1], Dario F. De Jesus[4], Laura Marroquí[2,5], Ramon Bosch[1,3,6], Eduard Montanya[2,7], Francesc Sureda[3], Andrea Tura[8], Andrea Mari[8], Rohit N. Kulkarni[4], Joan Vendrell[1-3,9], Sonia Fernández-Veledo[1-3,9].

[1] Unitat de Recerca, Hospital Universitari Joan XXIII, Institut d'Investigació Sanitària Pere Virgili, Tarragona, 43005, Spain.

[2] CIBER de Diabetes y Enfermedades Metabólicas Asociadas (CIBERDEM), Instituto de Salud Carlos III, Madrid, 28029, Spain.

[3] Universitat Rovira i Virgili, Tarragona, 43003, Spain.

[4] Islet Cell and Regenerative Biology, Joslin Diabetes Center, Department of Medicine, Beth Israel Deaconess Medical Center, Harvard Stem Cell Institute, Harvard Medical School, Boston, MA, 02215, USA.

[5] Instituto de Investigación, Desarrollo e Innovación en Biotecnología Sanitaria de Elche (IDiBE), Universidad Miguel Hernández de Elche, Alicante, 03202, Spain.

[6] Histological, Cytological and Digitization Studies Platform, Pathology Department, Hospital Verge de la Cinta, Tortosa, 43500, Spain, and Hospital Universitari Joan XXIII, Tarragona, 43005, Spain.

[7] Hospital Universitari de Bellvitge, IDIBELL, and Universitat de Barcelona, Barcelona, 08908, Spain.

[8] Institute of Neuroscience, National Research Council, Padua, 35127, Italy.

[9] Co-senior authors: Sonia Fernández-Veledo and Joan Vendrell.

Corresponding author: Sonia Fernández-Veledo; Institut d'Investigació Sanitària Pere Virgili, C/ Dr. Mallafrè Guasch, 4, 43005, Tarragona, Spain; +34 977 295 800 ext. 3401; sonia.fernandez@iispv.cat.

SUPPLEMENTAL METHODS

Human islets

Human islets for gene expression analysis were isolated from the pancreas of non-diabetic adult brain-dead organ donors at Hospital Universitari de Bellvitge and Institut d'Investigació Biomèdica de Bellvitge (Barcelona, Spain) as previously described (1). The characteristics of the donors is listed in **Supplemental Table 1**.

Human islets for protein expression assays were obtained from the Integrated Islet Distribution Program and Prodo Laboratories (Aliso Viejo, CA, USA) and processed as previously detailed (2). Donor details are outlined in **Supplemental Table 2**.

FACS purification and cell culture of rat α- and β-cells

Rat islets were isolated by collagenase digestion and handpicked. Islets were dissociated into single cells by mechanical and enzymatic dispersion using trypsin (1 $mg \cdot ml^{-1}$; Sigma-Aldrich, Saint Louis, MO) and DNase I (1 $mg \cdot ml^{-1}$; Roche, Basel, Switzerland) for 5 min at 31 °C under agitation. Dissociated cells were resuspended in Earle's medium (0.79 mM NaH_2PO_4, 0.8 mM $MgSO_4$, 2.4 mM $CaCl_2$, 115 mM NaCl, 5.4 mM KCl) containing 2.8 mM glucose. FACS sorting of β- and α-cells was done using a FACSAria III cell sorter (BD Biosciences, Franklin Lakes, NJ), as described (3). The percentage of viability was determined after incubation with the DNA-binding dyes propidium iodide (5 $\mu g \cdot ml^{-1}$; Sigma-Aldrich) and Hoechst 33342 (5 $\mu g \cdot ml^{-1}$; Sigma-Aldrich). A minimum of 600 cells were counted in each experimental condition. Viability was evaluated by two independent observers, one of them blinded to sample identity. The agreement between observers was > 90% (3). After sorting, purified β-cells were cultured in Ham's F-10 medium containing 10 mM glucose, 2 mM GlutaMAX (Thermo Fisher Scientific, Waltham, MA), 0.5% bovine serum albumin fraction V (BSA; Sigma-Aldrich, Saint Louis, MO) 50 µM 3-isobutyl-1-methylxanthine (Sigma-Aldrich), 50 $U \cdot ml^{-1}$ penicillin, 50 $\mu g \cdot ml^{-1}$ streptomycin (Thermo Fisher Scientific) and 5% heat-inactivated fetal bovine serum (FBS; Thermo Fisher Scientific); α-cells were cultured in the same medium but including 6.1 mM glucose and 10% FBS. The purity of the α and β-cell preparations was evaluated by immunofluorescence and was calculated as a percentage of positive cells for each cell type (4). α- and β-cells were immunostained with mouse monoclonal anti-insulin or anti-glucagon antibodies (both from Sigma-Aldrich) for 1 h followed by incubation with a rabbit anti-mouse secondary antibody conjugated with Alexa Fluor 488 (A32723) or Alexa Fluor 568 (A11031) from (Thermo Fisher Scientific).

Mouse islet isolation

Pancreatic islets were isolated from 16-week-old male mice using collagenase digestion and subsequently handpicked following density gradient separation with Histopaque, as outlined previously (5). In brief, the pancreas was perfused with Collagenase P (1.8 $U \cdot mg^{-1}$ lyophilized;

Roche) through intraductal injection and then subjected to digestion in a water bath at 37 °C with gentle agitation. Following several washes with Hank's Balanced Salt Solution (Thermo Fisher Scientific) containing 0.1% BSA (HBSS-BSA), islets were separated from the upper phase of a density gradient using Histopaque with densities of 1.119 $g \cdot l^{-1}$ and 1.077 $g \cdot l^{-1}$ (Sigma-Aldrich), and then resuspended in HBSS-BSA. The isolated islets were handpicked and subsequently incubated overnight in RPMI 1640 medium (Thermo Fisher Scientific) at 37 °C with 5% CO_2 prior to GSIS assays.

Pancreas histology

Pancreatic tissue was obtained from the autopsy of an adult human male and from 16-week-old male mice. Tissues were washed with PBS and fixed in 4% paraformaldehyde (Sigma-Aldrich) overnight at 4 ºC. Tissue samples were then dehydrated and degreased before paraffin embedding.

For immunohistochemistry, formalin-fixed paraffin blocks of pancreatic tissue were sectioned at a thickness of 4 µm, and each slide was deparaffinized in xylene for 20 min, rehydrated with a decreasing ethanol series and washed with PBS. Sections were heated at 96 °C for 20 min and then incubated for 30 min with a primary antibody against SUCNR1 (1:2 dilution, NLS3476; Novus Biologicals, Centennial, CO) (6) and chromogranin A (Clone DAK-A3; Agilent, Santa Clara, CA). Automatic immunodetection was performed using the EnVision FLEX method (Agilent) with 3,3'-diaminobenzidine chromogen as substrate and hematoxylin counterstain. Pannoramic 250 Flash III DX Scanner and SlideViewer 2.6 software (3DHISTEC, Budapest, Hungary) were used for image scanning, visualization, and image acquisition of stained pancreatic tissues (6).

For morphometric analysis, 3 non-consecutive (200 µm apart) 3-µm-thick sections were obtained and analyzed per mouse. Sections were subjected to H&E staining or immunostaining. For immunostaining, slides were heated, in 10 mM sodium citrate, followed by blocking with donkey serum and incubation with primary antibodies detecting insulin (sc-8033, Santa Cruz Biotechnology, Dallas, TX) and glucagon (SAB4501137; Sigma-Aldrich). Specific signals were detected with fluorochrome-conjugated secondary antibodies with Alexa Fluor 488 (A11029) or Alexa Fluor 568 (A21043) from Thermo Fisher Scientific. Slides were counterstained with DAPI (Sigma-Aldrich) to visualize nuclei. Images were captured using either a PANNORAMIC 250 Flash III brightfield scanning microscope (3DHISTECH Ltd., Budapest, Hungary) or a Zeiss Axio Vert A1 fluorescence microscope system (Zeiss, Oberkochen, Germany). Islet mass was assessed by calculating the ratio of the cross-sectional area of the total number of pixels from identified islets on H&E-stained slides, multiplied by the pancreas weight of the mouse. Similarly, β-cell mass was estimated by dividing the cross-sectional area of insulin-positive cells, as identified through immunostaining, by the total pancreatic tissue area indicated by serial H&E staining, incorporating the pancreas weight in the calculation. α-cell mass was calculated using an analogous approach but using the total number of pixels from glucagon-positive cells. Images were processed and analyzed using Fiji 2.9.0 software.

Succinate secretion assay and quantification

MIN6 cells were washed twice in Krebs-Ringer-Phosphate-HEPES (KRPH) buffer (5 mM Na_2HPO_4, 1 mM $MgSO_4$, 1 mM $CaCl_2$, 136 mM NaCl, 4.7 mM KCl, 20 mM HEPES pH 7.4, and 0.5% BSA), and then incubated for 1 h in KRPH buffer supplemented with 2.8 mM glucose at 37 °C with 5% CO_2. The buffer was then replaced with KRPH buffer containing 2.8 or 16.7 mM glucose, for 2 h at 37 °C with 5% CO_2. The CM was collected at the end of the assay was centrifuged at 500 RCF for 15 min at 4 °C and the supernatant was used to quantify succinate content using the fluorometric EnzyChrom Succinate Assay Kit (BioAssay Systems).

References

1. Nacher M, et al. Human Serum Versus Human Serum Albumin Supplementation in Human Islet Pretransplantation Culture: In Vitro and In Vivo Assessment. *Cell Transplant*. 2016;25(2):343–352.
2. De Jesus DF, et al. m6A mRNA methylation regulates human β-cell biology in physiological states and in type 2 diabetes. *Nat Metab*. 2019;1(8):765–774.
3. Marroqui L, et al. Pancreatic α Cells are Resistant to Metabolic Stress-induced Apoptosis in Type 2 Diabetes. *EBioMedicine*. 2015;2(5):378–385.
4. Marroqui L, et al. Differential cell autonomous responses determine the outcome of coxsackievirus infections in murine pancreatic α and β cells. *Elife*. 2015;4.
5. Díaz-Catalán D, et al. BACE2 suppression in mice aggravates the adverse metabolic consequences of an obesogenic diet. *Mol Metab*. 2021;53:101251.
6. Terra X, et al. Succinate Pathway in Head and Neck Squamous Cell Carcinoma: Potential as a Diagnostic and Prognostic Marker. *Cancers (Basel)*. 2021;13(7).

Supplemental Table 1. Characteristics of the islet donors (related to Figures 2B and 2D)

	Donor ID	Gender	Age (years)	BMI (kg·m^{-2})	HbA1c (%)	Islet Purity (%)
**Healthy**	94/14	Female	32	21.8	n/a	90
	100/15	Male	41	24.7	n/a	80
	101/15	Female	51	22.4	n/a	90
	106/15	Male	80	20.8	n/a	85
	109/16	Female	61	20.6	5.2	80
	119/17	Male	51	29.4	n/a	85
	134/19	Male	48	29.3	4.8	90
**Obesity**	97/14	Male	54	35.8	n/a	95
	104/15	Male	62	31.3	5.4	85
	110/16	Male	71	30.5	5.2	85

Abbreviations: Body mass index (BMI), hemoglobin A1c (HbA1c).

Supplemental Table 2. Characteristics of the islet donors (related to Figures 2C and 2D)

	Donor ID	Gender	Age (years)	Ethnicity	BMI ($kg \cdot m^{-2}$)	HbA1c (%)	Purity (%)	Viability (%)
**Healthy**	HP-17031-01	Female	64	Hispanic/Latino	24.6	5.4	95	95
	UNOS ADJV268	Male	39	Caucasian	23.7	n/a	90	90
	UNOS ADIY035	Male	15	Hispanic/Latino	24.6	n/a	80	97
	HP-17036-01	Male	59	Caucasian	26.9	5.8	90	90
	HP-20199-01	Male	21	Hispanic/Latino	27.3	5.1	90	95
	MGH (n/a)	Male	38	n/a	28.3	n/a	90	90
**Obesity**	RRID 12292085	Male	20	Hispanic/Latino	35.8	n/a	95	95
	UNOS ACEY097	Male	34	Caucasian	31.2	5.1	85	85
	HP-20206-01	Male	22	Hispanic/Latino	31.9	5.4	85	95
**Type 2 Diabetes**	UNOS ADHF167	Female	52	Hispanic/Latino	39.9	7.4	95	95
	HP-19078-T2D	Female	66	Caucasian	30.3	6.5	85	95
	HP-19171-T2D	Female	66	Hispanic/Latino	29.2	7.2	85	95
	RRID 12597653	Female	42	Hispanic/Latino	40.3	9.5	80	80
	HP-19131-T2D	Male	58	Caucasian	32.7	6.7	85	95
	HP-18320-T2D	Male	61	African American	27.4	7.1	85	95
	UNOS ADAHX156	Male	57	Hispanic/Latino	34.6	10.4	98	80

Abbreviations: Body mass index (BMI), hemoglobin A1c (HbA1c).

Supplemental Table 3. PCR primers and qPCR probes utilized in the study

qPCR TaqMan probes	Vendor	Catalog number
Human *SUCNR1*	Thermo Fisher Scientific	Hs00908230_m1
Human *GLP1R*	Thermo Fisher Scientific	Hs00157705_m1
Human *TBP*	Thermo Fisher Scientific	Hs00427620_m1
Rat *Sucnr1*	Thermo Fisher Scientific	Rn02084929_s1
Rat *B2m*	Thermo Fisher Scientific	Rn00560865_m1
Mouse *Sucnr1*	Thermo Fisher Scientific	Mm02620543_m1
Rat *B2m*	Thermo Fisher Scientific	Rn00560865_m1
Mouse *B2m*	Thermo Fisher Scientific	Mm00437762_m1
Mouse *18s*	Thermo Fisher Scientific	Mm04277571_s1
**PCR primers**	**Vendor**	**Sequence**
Mouse *Ins1* common forward	Integrated DNA Technologies	5'-GGCCAAACAGCAAAGTCCAG-3'
Mouse *Ins1* wild-type allele reverse	Integrated DNA Technologies	5'-GATCCACAATGCCACGCTTC-3'
Mouse *Ins1* Cre knockin allele reverse	Integrated DNA Technologies	5'-AACCAGCGTTTTCGTTCTGC-3'
Mouse *Sucnr1* forward	Integrated DNA Technologies	5'-AGACAAGTTATGACTATATGCTGAGAC-3'
Mouse *Sucnr1* reverse	Integrated DNA Technologies	5'-TTCACATCTATAATATAGCACCCTGTA-3'

Abbreviations: Succinate receptor 1 (*SUCNR1*), glucagon-like peptide 1 receptor (*GLP1R*), TATA-box binding protein (*TBP*), beta-2 microglobulin (*B2m*), 18S ribosomal RNA (*18s*), cyclization recombinase (*Cre*), insulin I (*Ins1*).

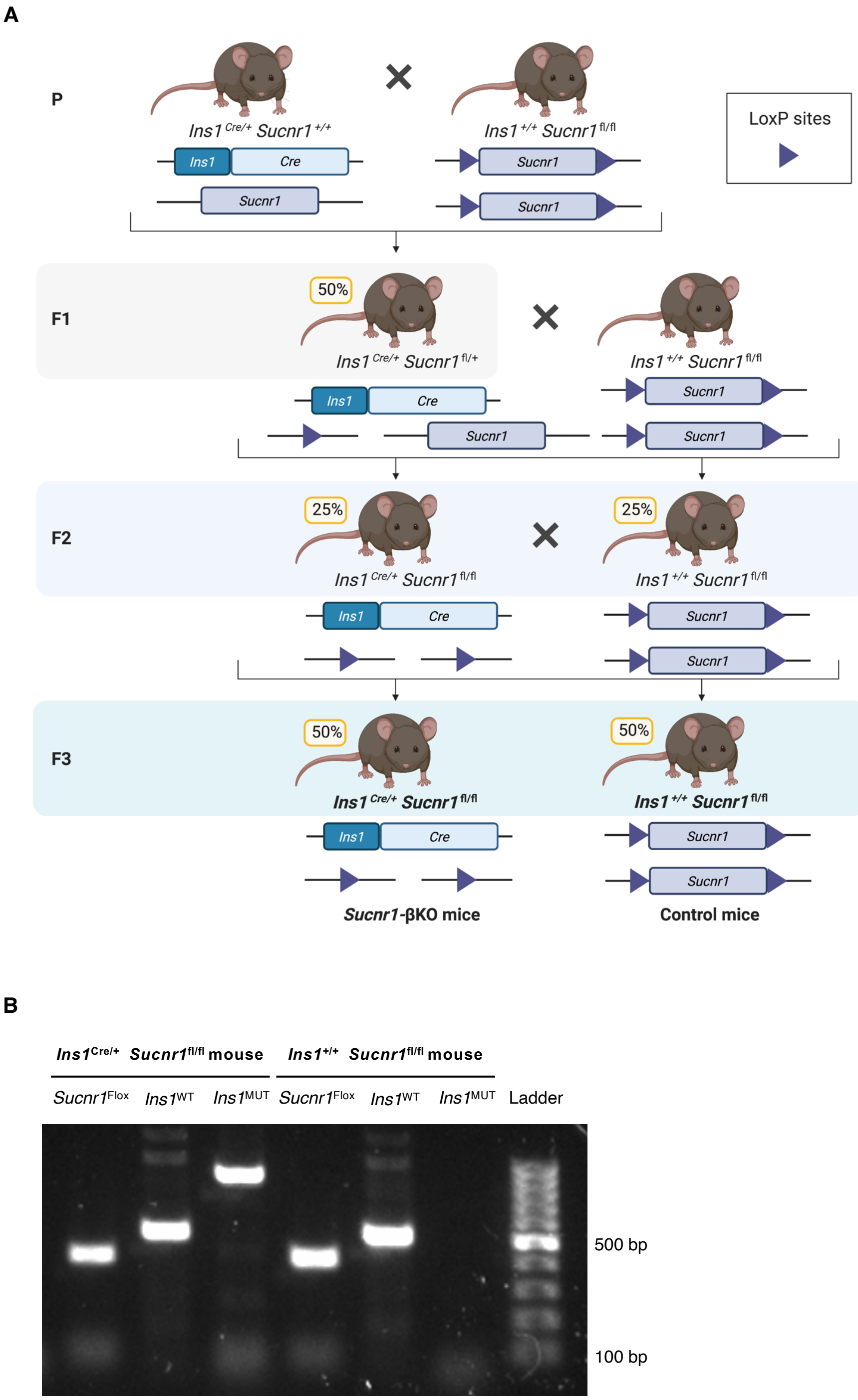

Supplemental Figure 1. Generation of β-cell-specific *Sucnr1* knockout mice and genotyping. (A) Breeding strategy to generate both control (*Ins1*$^{+/+}$ *Sucnr1*$^{fl/fl}$) and *Sucnr1*-βKO (*Ins1*$^{Cre/+}$ *Sucnr1*$^{fl/fl}$) mouse mice for the present study. **(B)** Genotyping of control and *Sucnr1*-βKO mice (*Sucnr1* floxed allele 450 bp; *Ins1* wild-type allele 524 bp; *Ins1* mutated allele 865 bp).

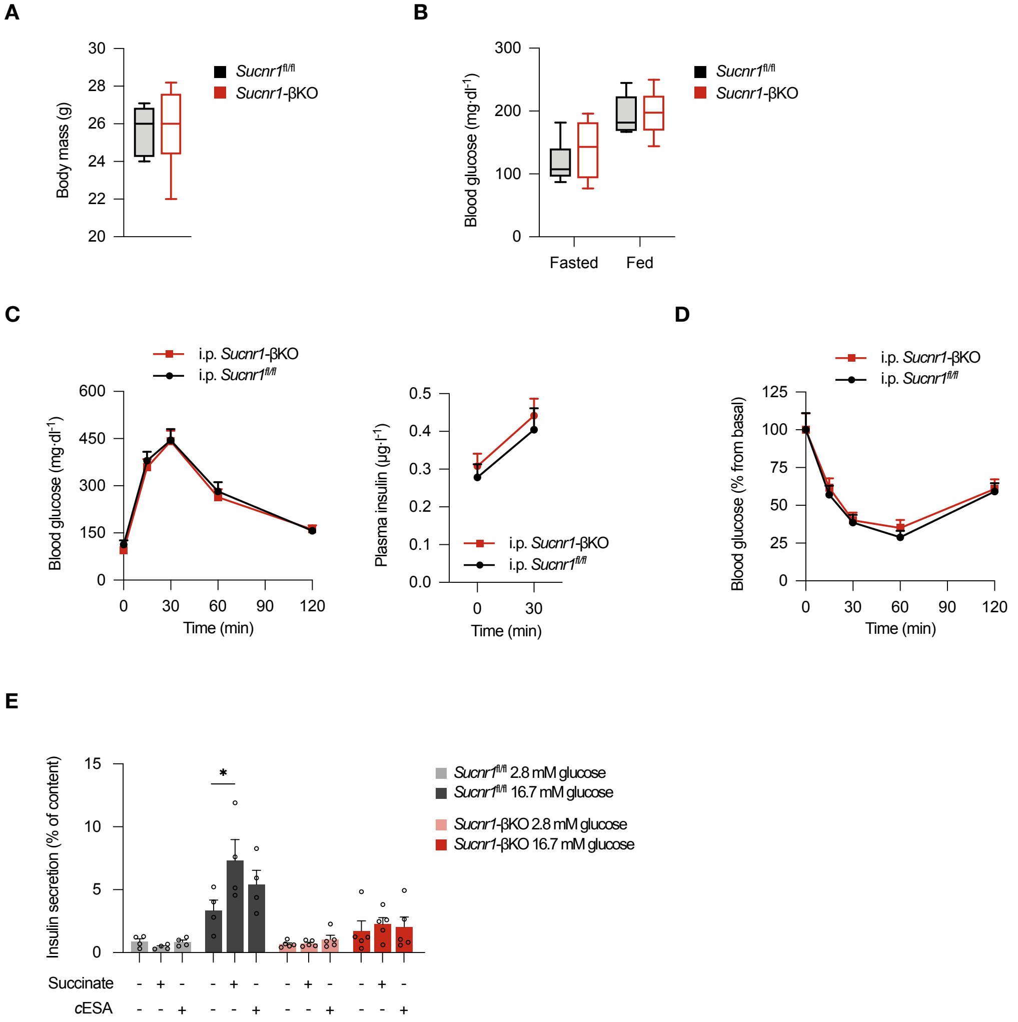

Supplemental Figure 2. *Sucnr1*-βKO mice under normal-chow diet (NCD) at 16 weeks of age. (A) Body mass of control and *Sucnr1*-βKO mice (n=8-10). **(B)** Blood glucose levels in control and *Sucnr1*-βKO mice in fasted or random-fed conditions (n=8-10). **(C)** Intraperitoneal glucose tolerance test in control and *Sucnr1*-βKO mice; displayed are blood glucose levels (n=8-10) and plasma insulin levels (n=6-7) during the test. **(D)** Insulin tolerance test in control and *Sucnr1*-βKO mice (n=8-10). **(E)** Insulin secretion in isolated islets from control and *Sucnr1*-βKO mice stimulated with or without 1 mM succinate or 100 µM *c*ESA at 2.8 or 16.7 mM glucose (n=4-5 islet pools from 4-5 mice). Data are displayed as mean ± SEM. **p*<0.05 (Two-way ANOVA with Bonferroni's test for multiple comparisons in **(E)**).

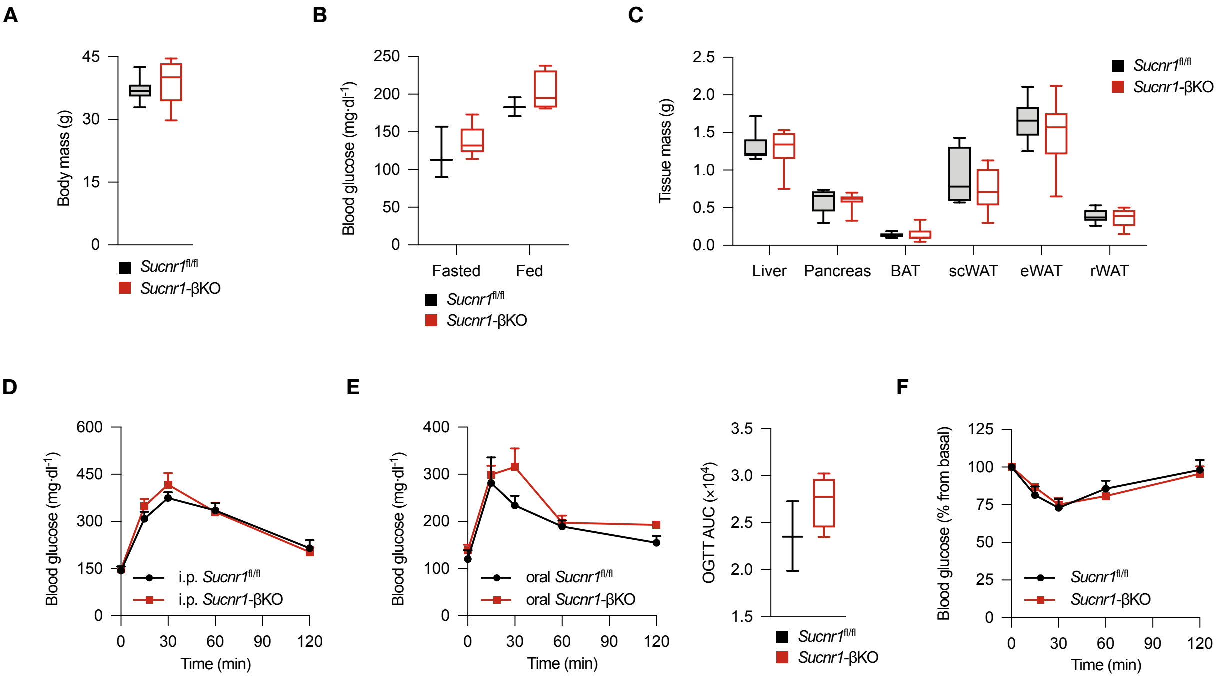

Supplemental Figure 3. Old-age *Sucnr1*-βKO mice under normal-chow diet (NCD). (A) Body mass of control and *Sucnr1*-βKO mice under NCD at 54 weeks (n=7-8). **(B)** Blood glucose levels in control and *Sucnr1*-βKO mice in fasted or random-fed conditions (n=3-5). **(C)** Tissue mass at the end of the study at 54 weeks (n=7-9). Brown adipose tissue (BAT), epididymal white adipose tissue (eWAT), and retroperitoneal white adipose tissue (rWAT). **(D)** Intraperitoneal glucose tolerance test in control and *Sucnr1*-βKO mice (n=7-9). **(E)** Oral glucose tolerance test in control and *Sucnr1*-βKO mice (n=3-4). **(F)** Insulin tolerance test in control and *Sucnr1*-βKO mice (n=7-9). Data are displayed as mean ± SEM.
