# Supplementary material for: SUCNR1 regulates insulin secretion and glucose elevates the succinate response in people with prediabetes
Source: J Clin Invest. 2024 May 7;134(12):e173214. doi: 10.1172/JCI173214 (PMC11178533; doi:10.1172/JCI173214)

Full unedited gels

Regarding the "chopping" of some immunoblots, we would like to clarify that we design each experiment so that we can perform the analysis of as many parameters as possible on precisely the very same membrane. We also aimed to avoid running multiple gels with limited samples, repeated stripping (thus minimizing stripping-derived interferences in Western Blot signals), and also diminish loading-derived differences since we test multiple proteins in exactly the same lane. This implies that sometimes each membrane is blotted with additional unrelated samples and several antibodies at the same time when this is allowed by the electrophoretic migration of the bands that are to be studied. The subsequent digital acquisition of the image is sometimes performed on the whole membrane but, for clarity, the image is then subdivided in as many sections as required for the number of proteins analyzed (thereby the "cropping" of the final image shown in the figure). Almost every membrane is treated in this way for the sake of reliability and reproducibility.

Full unedited gels for Figure 2C

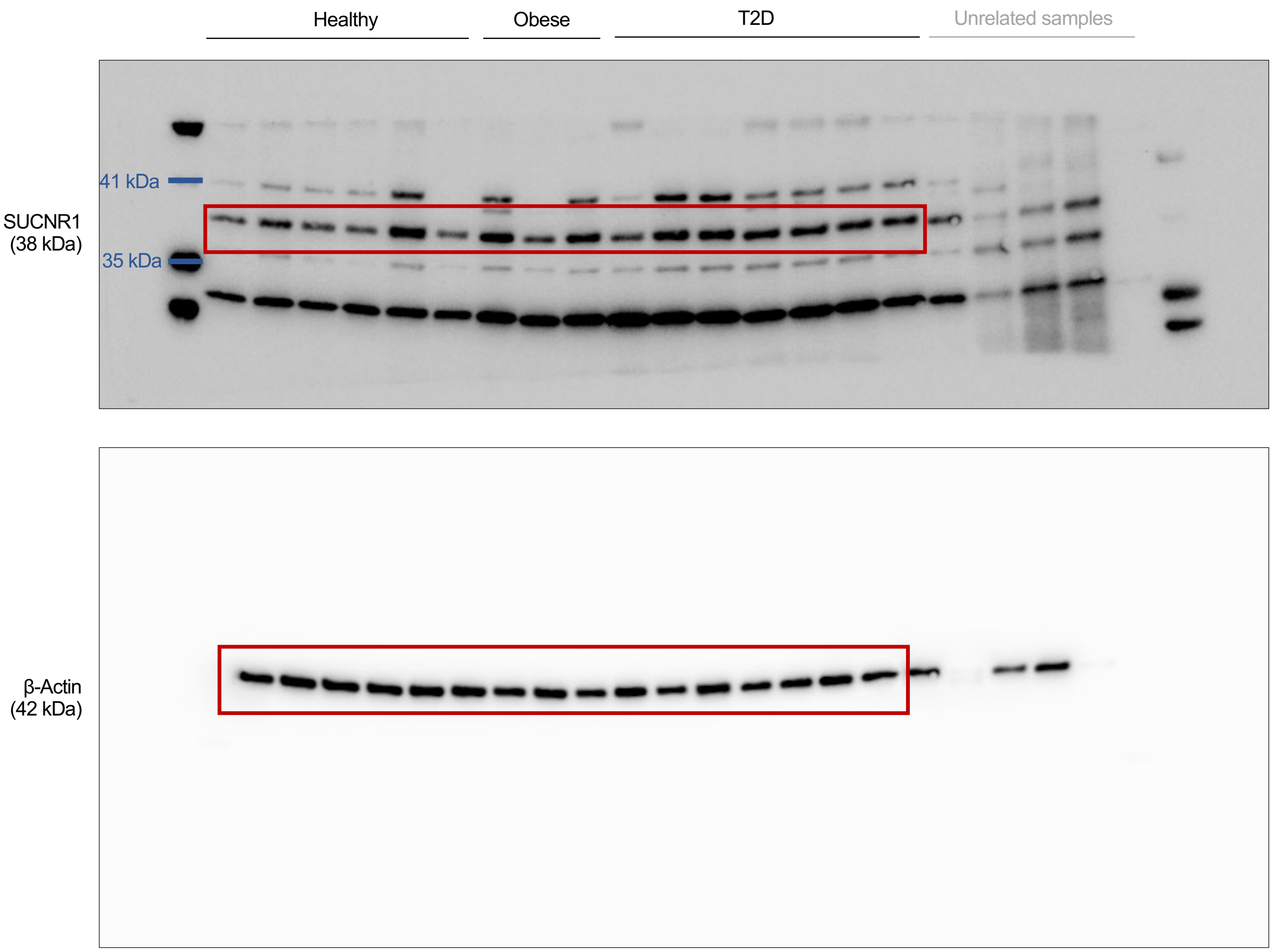

Full unedited gels for Figure 3C

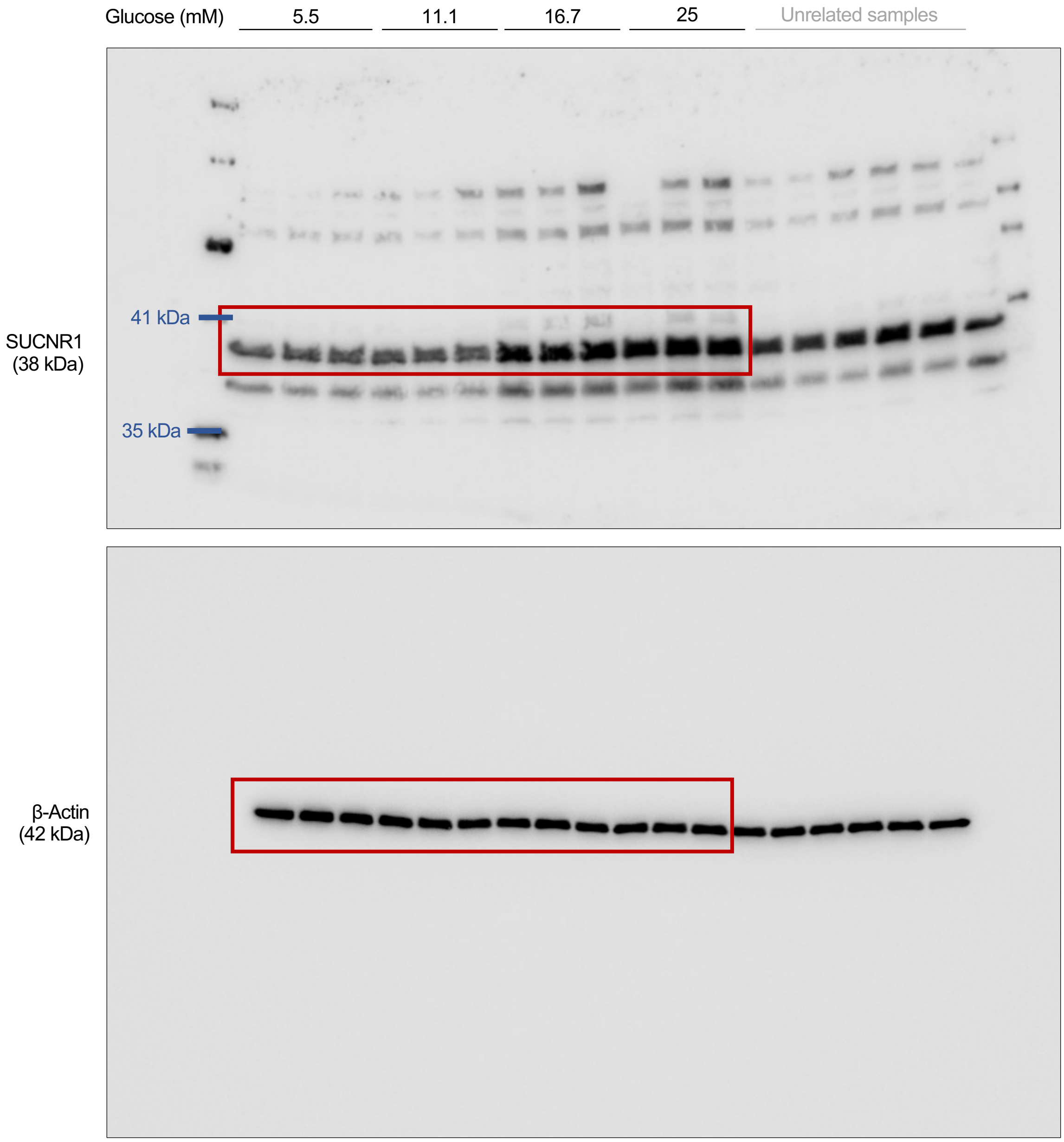

Full unedited gels for Figure 4A

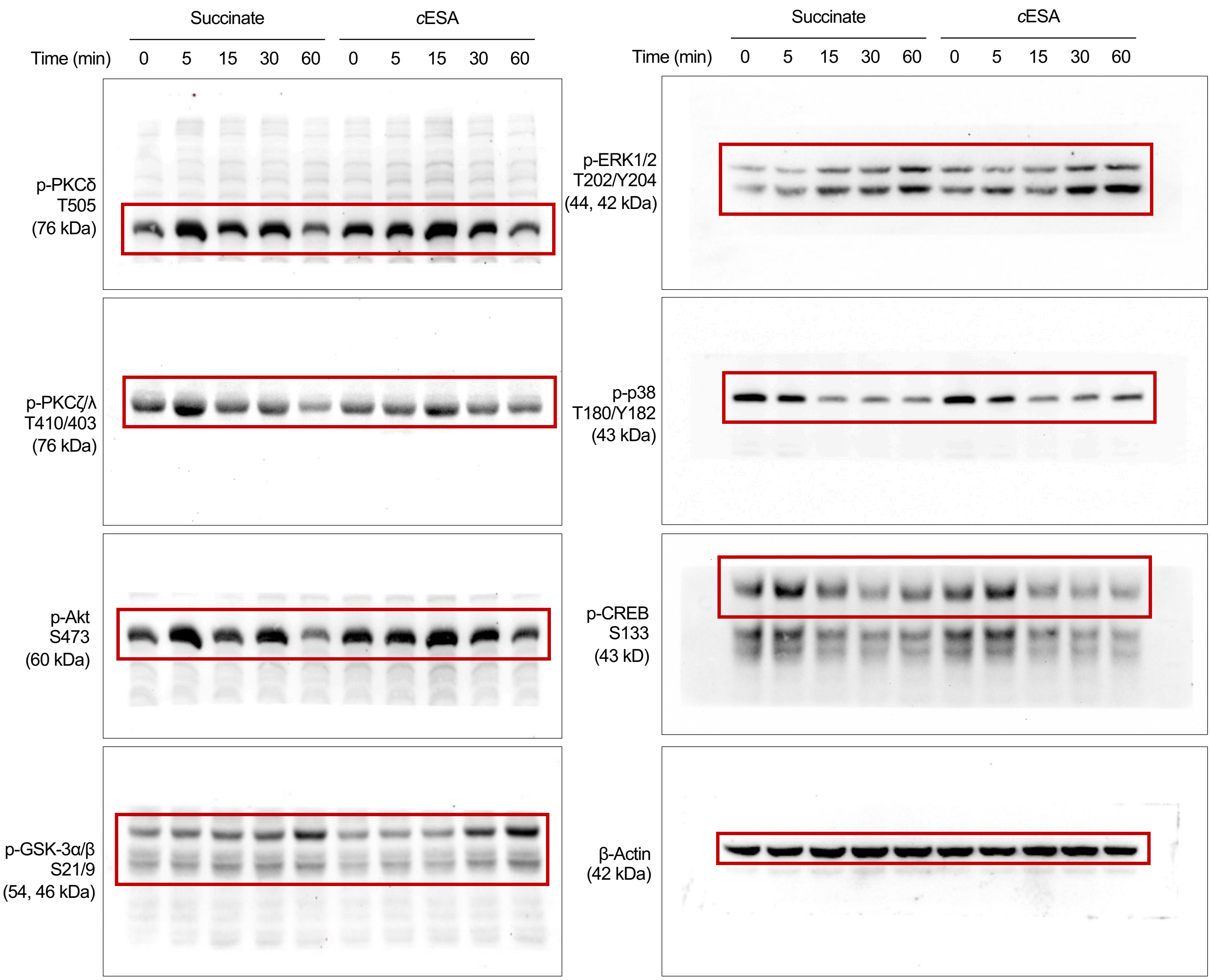

Supplement: Unedited blot and gel images [file jci-134-173214-s143.pdf]
